# Efficacy of new immunomodulatory drugs on major adverse cardiovascular events in patients with coronary heart disease: a systematic review and meta-analysis of randomized controlled trials

**DOI:** 10.1186/s12872-025-05250-1

**Published:** 2025-11-10

**Authors:** Donghang He, Yuhan Li, Zefei Jiang, Xin Cao, Rong Luo

**Affiliations:** 1 School of Medical and Life Sciences, Chengdu University of Traditional Chinese Medicine, Chengdu, Sichuan 611137 China; 2 School of Acupuncture-Moxibustion and Tuina, Chengdu University of Traditional Chinese Medicine, Chengdu, China; 3 Department of Acupuncture, Kunming Municipal Hospital of Traditional Chinese Medicine, The Third Affiliated Hospital of Yunnan University of Chinese Medicine, Kunming, Yunnan Province 650000 People’s Republic of China; 4 Key Laboratory of Acupuncture for Senile Disease, Acupuncture and Tuina School, Ministry of Education, Chengdu University of Traditional Chinese Medicine, 1166 Liutai Avenue, Wenjiang District, Chengdu, Sichuan 611137 China; 5 Institute of Geriatric Cardiovascular Disease, Chengdu Medical College, Chengdu, People’s Republic of China

**Keywords:** Coronary heart disease, New immunomodulatory drugs, Major adverse cardiovascular events, Meta-analysis, Systematic review

## Abstract

**Background:**

Despite optimal standard therapy, residual inflammation continues to increase major adverse cardiovascular events (MACE) in patients with coronary heart disease (CHD). New immunomodulatory drugs targeting specific immune pathways have shown mixed efficacy across trials, warranting comprehensive evaluation of their role in secondary prevention.

**Methods:**

We performed a systematic review and meta-analysis of 25 randomized controlled trials (RCTs) from January 1, 2014, to October 1, 2024, identified from eight databases: the cochrane library, (public medicine) pubmed, embase, web of science, china national knowledge infrastructure (CNKI), wanfang data knowledge service platform(WanFang), Weipu information database(VIP), and china biomedical literature database (SinoMed). Eligible studies assessed the efficacy of immunomodulatory agents, including colchicine, and canakinumab on MACE. Primary outcome was MACE incidence; secondary outcomes included, angina, and inflammatory biomarkers. Risk ratios (RR) with 95% confidence intervals (CI) were pooled using fixed or random-effects models. Subgroup analyses were conducted by drug class, follow-up duration, and CHD subtype (acute vs. chronic coronary syndrome). Risk of bias was assessed via Cochrane RoB 1.0, and evidence certainty rated with GRADE.

**Results:**

Overall, new immunomodulatory drugs did not significantly reduce MACE (RR = 0.92; 95% CI: [0.84,1.01]; *P* = 0.09; I²=60%). However, subgroup analyses revealed heterogeneous effects across drug classes. Significant reductions in MACE were observed with NLRP3 inflammasome inhibitors (RR = 0.75; 95% CI: 0.65,0.86; *P* < 0.0001) and interleukin-pathway inhibitors (RR = 0.86; 95% CI: 0.75,0.97; *P* = 0.02). In contrast, no significant reduction in MACE incidence was found in the broad-spectrum immunomodulator group, Lp-PLA2 inhibitor group, or p38 MAPK kinase inhibitor group (all *P* > 0.05). Besides, benefits were evident only in trials with follow-up exceeding 6 months (RR = 0.89; 95% CI: [0.82,0.98]. Secondary outcomes showed significant reductions in angina (RR = 0.72; 95%CI: [0.58,0.90], *P* = 0.004), revascularization (RR = 0.85; 95%CI: [0.73,0.98], *P* = 0.03), IL-6 (SMD = − 0.82;95༅CI: [-1.62,-0.03], *P* = 0.02), and neutrophil count, but no effect on (cardiac arrest)CA, all-cause mortality, incidence of gastrointestinal adverse effect and high-sensitivity c-reactive protein(hs-CRP). The quality of evidence for MACE was assessed as moderate.

**Conclusion:**

Targeted anti-inflammatory therapies, particularly colchicine and canakinumab, significantly reduce MACE in CHD patients when used for longer than six months. Efficacy varies by mechanism of action, supporting precision use of NLRP3 and IL-1β inhibitors. Future trials should been focus on biomarker-guided, long-term anti-inflammatory interventions in cardiovascular care.

**Trial Registration:**

https://www.crd.york.ac.uk/PROSPERO/view/CRD42024597008PROSPERO: CRD42024597008.

**Supplementary Information:**

The online version contains supplementary material available at 10.1186/s12872-025-05250-1.

## Introduction

Cardiovascular death remains the leading cause of mortality worldwide. According to 2020 mortality data, heart disease and stroke now cause more deaths each year than the combined total of cancer and chronic lower respiratory diseases. In 2020, the death rate from heart disease and stroke was 207.1 per 100,000 people. In 2020, cardiovascular diseases (CVD) were estimated to account for 19.05 million deaths worldwide, representing an increase of 8.71% compared to 2010. Ischemic heart disease climbed from the third leading cause of death in 1990 to the top cause by 2019 [1].

Coronary heart disease (CHD) patients remain at high risk of major adverse cardiovascular events (MACE), including myocardial infarction, cardiovascular death, and cardioembolic stroke, despite receiving optimized, guideline-directed therapies. The recurrence of MACE remains concerningly high: antiplatelet therapy is associated with a recurrence rate of up to 27.7%, while statin therapy and even complete revascularization do not fully mitigate this risk, with over 20.0% of patients experiencing recurrent events within three years [2].

Repeated hospitalizations, reduced functional capacity, and increasing financial stress place a dual physical and psychological burden on affected individuals. Therefore, identifying more effective therapeutic strategies to improve long-term outcomes in CHD has become a critical public health priority.

Contemporary CHD management strategies are based on three pillars: thrombus inhibition (e.g., aspirin), reduction of low-density lipoprotein cholesterol (e.g.,statins), and restoration of coronary blood flow perfusion (e.g.,stenting) [3]. However, emerging evidence suggests atherosclerosis is fundamentally a chronic inflammatory process [4]. Even under optimal lipid-lowering and antithrombotic therapy, the residual inflammation risk contribute to drive adverse cardiovascular events [5]. The landmark Canakinumab Anti-Inflammatory Thrombosis Outcomes Study (CANTOS) first demonstrated that targeted inhibition of interleukin-1β(IL-1β) significantly reduced MACE risk independently of lipid levels [6]. However, the subsequent trials have yielded mixed results: low-dose colchicine significantly reduced MACE in Colchicine Cardiovascular Outcomes Trial (COLCOT) and Low-Dose Colchicine 2 trial (LoDoCo2) [7, 8], whereas methotrexate failed to confer cardiovascular benefits in the Cardiovascular Inflammation Reduction Trial (CIRT) [9].These findings underscore the persistent controversy regarding the clinical utility of new immunomodulatory drugs. Evidence for the efficacy and safety of targeting alternative inflammatory mediators, such as IL-6, TNF-α, and the NLRP3 inflammasome, remains inconsistent, contributing to uncertainty in guideline recommendations. A comprehensive evaluation of these agents is thus crucial for refining secondary prevention strategies in CHD.

Previous meta-analyses focused narrowly on individual agents, such as colchicine [10], and often excluded more recent trials. To address this gap, we conducted a systematic review and meta-analysis of updated RCT evidence to address the following key questions: (1) Do inflammatory pathway inhibitors reduce MACE risk in patients with CHD? (2) Are there differences in efficacy and safety among drug classes or molecular targets? (3) Is existing evidence sufficient to guide precision medicine approaches in targeted CHD populations, including stable angina and acute myocardial infarction cohorts?

By addressing these questions, our study aims to provide robust evidence to guide clinical decision-making and providing a theoretical foundation for the development of immunomodulatory approaches in cardiovascular medicine.

### Overview and registration

This meta-analysis was conducted in accordance with the recommendations of the “Preferred Reporting Entries for Systematic Reviews and Meta-Analyses” (PRISMA) statement and has been registered in the PROSPERO database of prospective Systematic Reviews (Registration ID: [CRD42024597008]).

### Search strategy

A comprehensive literature search was performed in PubMed, Embase, Cochrane Library, Web of Science, Sinomed, VIP Information, Wanfang Data Knowledge Service Platform (WanFang), and China National Knowledge Infrastructure (CNKI) from January 1, 2014, to October 1, 2024, without language restrictions. The search strategy combined terms for (1) new immunomodulatory drugs, such as interleukins, colchicine and methotrexate. (2) coronary artery disease, using Medical Subject Headings (MeSH) and free-text keywords adapted for each database. Supplementary Table 1 lists the adjusted search strategies for each database. Conduct manual searches of relevant conference abstracts, clinical trial registries (e.g., ClinicalTrials.gov), and perform citation tracing of reference literature.

### Inclusion criteria

(1)Population: Adult patients (≥ 18 years) with a diagnosis of coronary heart disease (CHD), as defined by recognized guidelines [11, 12], confirmed by one or more of the following objective criteria: a history of myocardial infarction (MI), percutaneous coronary intervention (PCI), coronary artery bypass grafting (CABG), significant multivessel coronary artery disease documented by angiography, or ischemic cardiomyopathy (left ventricular ejection fraction < 35% of ischemic etiology).(2) Intervention: use of new immunomodulatory drugs. (3) Comparison: placebo or other drugs. (4) Outcome: studies with cardiovascular outcomes (e.g., MACE, Stroke). (5) Study Design: randomized controlled trials (RCTs). (6) Publication Date: Studies published from January 2014 to October 2024. (7) Data Availability: full-text accessible with complete outcome data.

### Exclusion criteria

1. Duplicate studies. (2) Studies with non-cardiovascular outcomes. (3) Non-randomized controlled studies. (4) No valid data was provided or the data was missing.

### Study selection

The retrieved literature was imported into Endnote 20, and duplicate literature was deleted. Two reviewers used the aforementioned inclusion and exclusion criteria to screen the studies by reading the titles and abstracts. Following the initial screening, the full texts of the identified studies were reviewed to determine their eligibility, and those that did not meet the inclusion criteria were excluded. Conflicting assessments of study eligibility were resolved through reviewer discussion or consultation with a third reviewer.

### Risk of bias assessment

The quality assessment of the included study was conducted in accordance with the standards outlined in the Cochrane Handbook of Systematic Reviews of Interventions (Edition 5.0.1). Two independent reviewers will use the Cochrane Risk of Bias Assessment Tool (RoB 2) to evaluate the risk of bias in randomized controlled trials. Each criterion was assessed using three rating categories based on the study specifics: “low risk”, “high risk”, or “unclear risk”. Any discrepancies will be resolved through discussion or arbitration by a third reviewer [13].

### Data extraction

This study included RCTs from 2014 to 2024 that explored the effects of various inflammatory pathway blockers on the cardiovascular system of patients with CHD. These drugs included anakinra, colchicine, tocilizumab, canakinumab, varespladib, darapladib, losmapimod, and methotrexate. The primary cardiovascular outcomes we focused on included MACE, all-cause mortality, revascularization, and several inflammatory indicators. MACE is defined as a composite of cardiovascular death, myocardial infarction, and stroke [14]. In studies that did not report the definition of MACE, we calculated the numbers of these three events based on the aforementioned definition to obtain this composite indicator. In studies employing multiple drug doses, we included them in separate groups. For studies reporting clinical outcomes at multiple follow-up time points, we utilized the data from the longest follow-up time. Two reviewers independently extracted data items from the included studies and assessed the eligibility of the studies based on the aforementioned criteria. The research began with a preliminary screening through titles and abstracts. Following the initial screening, a comprehensive review of the confirmed research texts was conducted to determine their eligibility. Baseline characteristics and clinical outcomes of interest were extracted using standardized data extraction tables. Some studies did not show the continuous data directly. For studies reporting continuous data as medians and interquartile ranges (IQRs), we estimated the means and standard deviations (SDs) using established statistical conversion formulas and incorporated these values into our literature database. Any discrepancies were resolved through discussion. A third reviewer will be consulted if required.

### Statistical analysis

In this study, data synthesis was performed using Review Manager 5.4 software (Cochrane Collaboration, Oxford, UK). The meta-analysis of categorical variables was conducted using the Mantel-Haenszel (M-H) statistical method to calculate risk ratios (RR) with 95% confidence intervals (CI). For continuous variables, the inverse variance (I-V) statistical method was employed to calculate mean differences (MD), standardized mean differences (SMD), and 95% CI. MD was used to interpret outcomes when the units and dimensions of the measurement scales were consistent (e.g., neutrophil count), while SMD was applied for outcomes with varying units (e.g., hs-CRP, IL-6). Statistical heterogeneity across pooled data was assessed using the I² statistic: an I²<50% indicated low heterogeneity, and a fixed-effect model (FEM) was selected for synthesis; an I²≥ 50% suggested substantial heterogeneity, in which case a random effects model (REM) was used to synthesize the data. All tests were two-tailed, and statistical significance was defined as *P* < 0.05. Conducting a meta-regression by using Stata 17.0 to explore potential sources of heterogeneity.

Subgroup analyses were performed based on the following three criteria: (1) Type of new immunomodulatory drugs (e.g., colchicine, methotrexate); (2) Follow-up duration (≤ 6 months vs.>6 months); (3) Disease classification of enrolled patients [ACS (Acute Coronary Syndrome) or CCS (Chronic Coronary Syndrome)]. Sensitivity analysis was conducted by gradually excluding the included individual studies to explore the impact of the quality of individual studies on the overall results. Publication bias was evaluated using funnel plots when ≥ 10 studies reported primary outcome data [13], meanwhile, Begg’s test and Egger’s test were conducted to calculate the publication bias [15]– [16].

This study utilized the GRADE (Grading of Recommendations Assessment, Development, and Evaluation) system to evaluate the quality of evidence for primary outcomes. The GRADE system reduced evidence quality based on five domains: risk of bias, inconsistency, indirectness, imprecision, and publication bias. Evidence quality was categorized into four levels: high, moderate, low, or very low. Two independent researchers conducted assessments using the GRADE pro GDT online tool, with disagreements resolved by discussion and consultation with a third reviewer.

## Result

### Search results and study characteristics

**Fig. 1:**
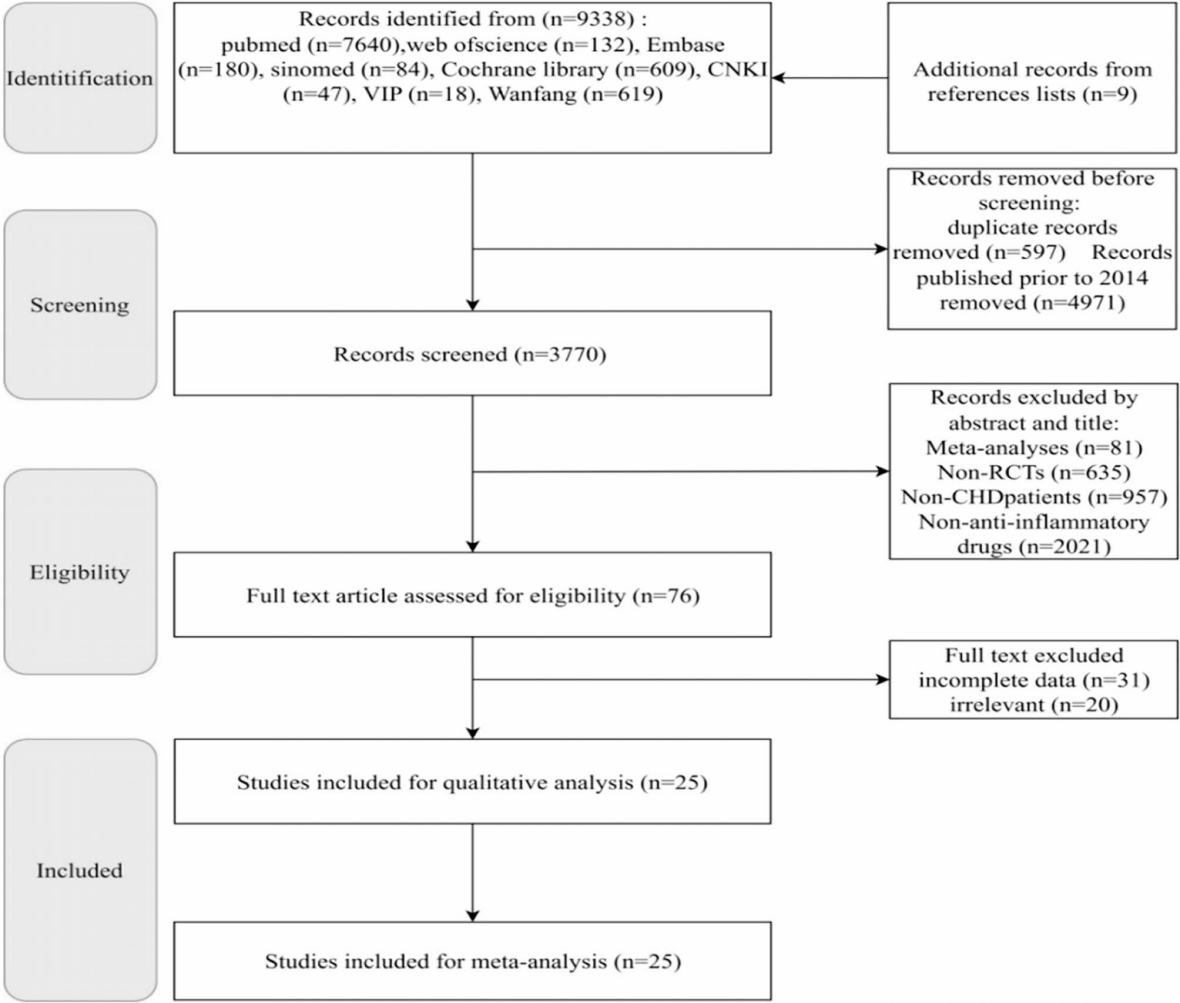
Flow chat

Initially, 9,329 relevant articles were retrieved from 8 databases, with an additional 9 articles identified from reference lists, totaling 9,338 articles. After excluding non-recent (older than 10 years) and duplicate publications, 3,770 articles remained for screening. Following title/abstract review and full-text assessment, a final total of 25 articles met the inclusion criteria [6–9, 14, 17–36]. The PRISMA flowchart is shown in Fig. 1.

The included studies comprised large-scale projects such as CANTOS and COLCOT, along with several smaller investigations. The new immunomodulatory drugs were utilized: anakinra (3 studies), colchicine (15 studies), tocilizumab (1 study), canakinumab (1 study), varespladib (1 study), darapladib (2 studies), methotrexate (1 study), and losmapimod (1 study). Baseline characteristics of the study populations are summarized in Table 1.

**Table 1:** Characteristics of studies

Number	Study ID	Country	Sample size(E/C)	Gender(F/M)	Age(E/C)	Intervention	Comparison	Type of drugs	Outcome	Duration	Type	Diagnosis methods	Funding sources
1	Abbate 2015 [17]	US	20/20	E:2/18 C:8/12	E:58 ± 11.2 C:54.8 ± 9.6	anakinra 100 mg QD	placebo	IL-pathway inhibitor	ACDE	28months	RCT	History of PCI	National Institutes of Health
2	Abbate 2020 [18]	US	E1:33 E2:31 C:35	E1:9/27 E2:5/26 C:5/30	E1:54.8 ± 10.1 E2: 53.6 ± 12.4 C: 57.4 ± 10.8	E1:anakinra 100 mg QD E2:anakinra 100 mg BID	placebo	IL-pathway inhibitor	A	12months	RCT	History of MI, CABG	National Institutes of Health
3	Akodad 2017 [19]	France	23/21	E:4/19 C:5/16	E:60.1 ± 13.1 C:59.7 ± 11.4	colchicine 1 mg QD	OMT alone	NLRP3 inflammasome inhibitor	AG	1month	RCT	History of MI, PCI	No
4	Akrami 2021 [20]	Iran	120/129	E:34/86 C: 42/87	E:56.9 ± 7.56 C:56.89 ± 7.45	colchicine 0.5 mg QD	placebo	NLRP3 inflammasome inhibitor	ACD	6 months	RCT	History of MI, significant multivessel coronary artery disease documented by angiography	Shiraz University of Medical Sciences
5	Broch 2021 [21]	Norway	101/98	E:21/80 C:11/87	E:62 ± 10 C:60 ± 9	tocilizumab 280 mg	0.9%NaCl 100 ml	IL-pathway inhibitor	GH	6 months	RCT	History of MI	South-Eastern Norway Regional Health Authority, the Central Norway Regional Health Authority, and Roche
6	Deftereos 2015 [22]	Greece	77/74	E:25/52 C:18/52	E:58 ± 9 C:59 ± 12.72	colchicine 0.5 mg BID	placebo	NLRP3 inflammasome inhibitor	GH	during hospitalization	RCT	History of MI	Investigator-initiated and -funded study.
7	Ridker 2017 [6]	America	E1:2170 E2:2284 E3:2263 C:3344	E1:541/1629 E2:572/1712 E3:606/1657 C:865/2479	E1:61.1 ± 10.1 E2:61.2 ± 10.0 E3:61.1 ± 10.1 C:61.1 ± 10.0	E1:canakinumab 50 mgQ3M E2:canakinumab 150 mgQ3M E3:canakinumab 300 mg Q3M	placebo	IL-pathway inhibitor	ACDE	3.7 years	RCT	History of MI	National Institutes of Health
8	Hennessy 2019 [23]	Australia	119/118	E:30/89 C:25/93	E:61 ± 13.6 C:61 ± 12.5	colchicine 0.5 mg QD	placebo	NLRP3 inflammasome inhibitor	G	1 month	RCT	History of MI	National Heart Foundation of Australia
9	Kajikawa 2019* [24]	Japan	14/14	1/27	68 ± 7	colchicine 0.5 mg QD	placebo	NLRP3 inflammasome inhibitor	G	14days	RCT	History of MI, PCI, CABG, significant multivessel coronary artery disease documented by angiography	Ministry of Health, Labour and Welfare
10	Martinez 2015 [25]	Australia	E1:40 E2:33 C:10	E1:3/37 E2:3/30 C:3/7	E1:64.5 ± 10.2 E2:61.1 ± 10. C:61.3 ± 6.7	E1:ACS with colchicine 1.5 mg E2:CAD with colchicine 1.5 mg	placebo	NLRP3 inflammasome inhibitor	G	during hospitalization	RCT	History of MI	Sydney Medical School Foun dation Grant
11	Mewton 2021 [26]	France	101/91	E:21/80 C:17/74	E:59.0 ± 10.6 C:60.9 ± 10.4	colchicine 0.5 mg BID	placebo	NLRP3 inflammasome inhibitor	ACHI	12 months	RCT	History of MI, PCI	French Ministry of Health
12	Morton 2014 [27]	UK	93/89	E:30/63 C:22/67	E:61.4 ± 11.7 C:61.3 ± 12.3	anakinra 100 mg QD	placebo	IL-pathway inhibitor	AFG	12 months	RCT	History of MI	UK Medical Research Council Experimental Medicine Grant
13	Nicholls 2014 [28]	Australia	2573/2572	E:660/1913 C:691/1881	E:60.7 ± 9.8 C:61.0 ± 10.0	varespladib 500 mg QD	placebo	PhospholipaseA2 inhibitor	ACD	4 months	RCT	History of MI	Anthera Pharmaceuticals.
14	Nidorf 2020 [8]	Australia	2762/2760	E:457/2305 C:389/2371	E:65.8 ± 8.4 C:65.9 ± 8.7	colchicine 0.5 mg QD	placebo	NLRP3 inflammasome inhibitor	ACD	29 months	RCT	Significant multivessel coronary artery disease documented by angiography	NHMRC project grant
15	O’Donoghue 2014 [29]	UK	6504/6522	E:1657/4847 C:1669/4853	E:64.4 ± 8.2 C:64.6 ± 8.9	darapladib 160 mg QD	placebo	PhospholipaseA2 inhibitor	ACDE	2.5years	RCT	History of MI	GlaxoSmithKline.
16	O’Donoghue 2016 [30]	UK	1731/1758	E:500/1231 C:532/1226	E:67.1 ± 9.6 C:67 ± 8.9	losmapimod 7.5 mg BID	placebo	P38MAPK kinase inhibitor	AE	6 months	RCT	History of MI	GlaxoSmithKline
17	Psaltis 2024 [31]	Australia	32/32	E:4/28 C:2/32	E:59.16 ± 11.22 C:64.42 ± 11.53	colchicine 0.5 mg QD	placebo	NLRP3 inflammasome inhibitor	G	18 months	RCT	History of MI, significant multivessel coronary artery disease documented by angiography	National Health and Medical Research Council
18	Ridker 2019 [9]	US	2391/2395	E:461/1930 C:437/1958	E:65.71 ± 8.97 C:65.83 ± 8.82	methotrexate 18.8 mg QW	placebo	Broad-spectrum immunomodulator	ADE	6 months	RCT	History of MI, significant multivessel coronary artery disease documented by angiography	National Institutes of Health
19	Roubille 2024 [32]	Canada	462/497	E:106/356 C:107/390	E:62.5 ± 10.4 C:62.4 ± 10.7	colchicine 0.5 mg QD	placebo	NLRP3 inflammasome inhibitor	ABEF	22.6 months	RCT	History of MI,	The Canadian Institutes of Health Research
20	Shah 2020 [33]	US	206/194	E:13/193 C:13/181	E:65.9 ± 9.9 C:66.6 ± 10.2	colchicine 1.8 mg	placebo	NLRP3 inflammasome inhibitor	ADEF	1 month	RCT	History of MI, PCI	American Heart Association Clinical Resea9rch Program
21	Tardif 2019 [7]	Canada	2366/2379	E:472/1894 C:437/1942	E:60.6 ± 10.7 C:60.5 ± 10.6	colchicine 0.5 mg QD	placebo	NLRP3 inflammasome inhibitor	ABEEF	23 months	RCT	History of MI,	No
22	Tong 2020 [34]	Australia	396/399	E:74/322 C:89/310	E:59.7 ± 10.2 C:60.0 ± 10.4	colchicine 0.5 mg BID for the first month, 0.5 mg QD for 11 months	placebo	NLRP3 inflammasome inhibitor	ACDE	12 months	RCT	History of MI, significant multivessel coronary artery disease documented by angiography	Peninsula Health and Faculty of Medicine
23	Vaidya 2018 [35]	Australia	40/40	E:8/32 C:10/30	E:56.3 ± 8.9 C:58.4 ± 14.2	colchicine 0.5 mg QD plus OMT	OMT alone	NLRP3 inflammasome inhibitor	G	12 months	RCT	Significant multivessel coronary artery disease documented by angiography	No
24	White 2014 [14]	UK	7904/7924	E: 1506/6398 C: 1461/6463	E:65 ± 8.9 C:65 ± 8.9	darapladib 160 mg QD	placebo	PhospholipaseA2 inhibitor	ACDE	3.7 years	RCT	History of MI, PCI, CABG, significant multivessel coronary artery disease documented by angiography	No
25	Xu 2023 [36]	China	E1:107 E2:108 C:95	E1:29/78 E2:30/7 C:20/75	E1:61.5 ± 12.2 E2:62.5 ± 12.2 C:60.5 ± 10.8	E1:colchicine 0.5 mg QD E2:colchicine 0.25 mg QD	placebo	NLRP3 inflammasome inhibitor	ACHGI	12 months	RCT	History of MI	Science and Technology Plan Project of Huadu District, Guangzhou City

A. MACE B. Cardiac arrest C. Unstable angina D. Any case of death E. Revascularization F. Gastrointestinal adverse reaction G. Inflammatory markers

*OMT* optimal medical therapy, *NLRP3* NOD-like receptor family, pyrin domain-containing protein 3 inflammasome, *IL* Interleukin, *P38MAPK* kinase inhibitor, p38 Mitogen-Activated Protein Kinase Inhibitor, *PCI* percutaneous coronary intervention, *LVEF* left ventricular ejection fraction, *CABG* coronary artery bypass grafting, *MI* myocardial infarction

H.Infarct size I.LVEF. *No age or gender grouping was performed (the literature did not provide grouped data)

### Risk of bias assessment

**Fig. 2:**
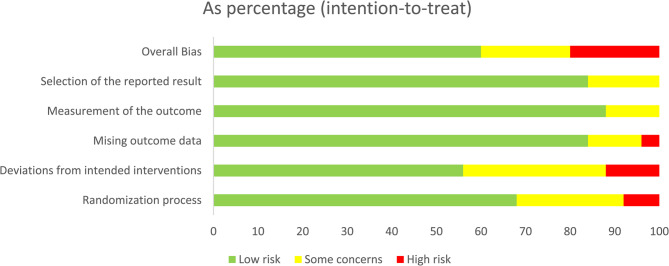
Risks of bias of the included studies. risk of bias. Green: low risk; yellow: some concern; red: high risk

This systematic review included a total of 25 RCTs, rigorously assessed using the Cochrane Risk of Bias tool (RoB 2.0). The risk of bias assessment results are illustrated in Fig. 2. Details information is shown in supplement Fig. 2.

Randomization process: most studies were judged as low risk, although a few raised some concerns, and two studies were judged as high risk [25, 35]. Deviations from intended interventions: 8 studies were rated as having some concerns [17]– [18, 25–27, 29, 35]– [14], and 3 studies were rated as high risk [19, 22, 36], primarily due to the open-label design without placebo control. Missing outcome data: most studies were rated as low risk because all randomized participants completed follow-up. One study was considered high risk due to a high rate of loss to follow-up [34]. Measurement of the outcome: The majority of studies were judged as low risk, particularly those employing blinded outcome assessment; only three studies were rated as having some concerns [19, 25, 36]. Selection of the reported result: Four studies raised some concerns, mainly due to unavailable preregistered protocols or potential selective reporting [23–25, 29].

### Primary outcome

**Fig. 3:**
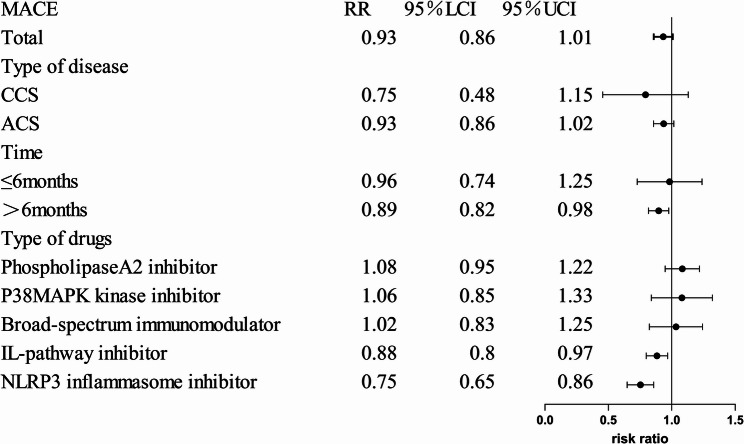
Subgroup analysis of MACE

The primary outcome of this study was the incidence of MACE. Subgroup analyses were conducted based on the type of new immunomodulatory drugs, follow-up duration, and disease classification, with results detailed in Fig. 3.

#### Overall summary

Among the 25 included studies, 15 reported the incidence of major adverse cardiovascular events (MACE) [6–9, 14, 17, 20, 26–30, 32–34]. Adding new immunomodulatory drugs to standard therapy during treatment did not show significant improvement in MACE incidence compared to standard therapy alone. (RR = 0.92,95%CI: [0.84,1.01], *P*=0.09, I²=60%, 17 trials, 65,420 participants).

#### Subgroup analysis based on the types of new immunomodulatory drugs

Based on different immunomodulatory drugs, these studies were divided into the IL-pathway inhibitors, NLRP3 inflammasome inhibitors, Lipoprotein-associated phospholipase A2 (Lp-PLA2) Inhibitors, p38 Mitogen-Activated Protein Kinase (MAPK) Inhibitors, broad-Spectrum immunomodulators. Compared with the control group, the incidence of MACE was significantly reduced in the NLRP3 inflammasome inhibitors group (RR = 0.75, 95% CI: [0.65,0.86], *P* < 0.0001), and the IL-pathway inhibitors group (RR = 0.86, 95% CI: [0.75,0.97], *P* = 0.02). However, in the Broad-spectrum immunomodulator group, Lp-PLA2 inhibitor group, and p38 MAPK kinase inhibitor group, no significant improvement in MACE incidence was observed compared with the control group (*P* > 0.05). The forest plot are shown in Supplement Fig. 2.1.

#### Subgroup analysis based on the follow-up time

Based on the follow-up periods, these studies were divided into a group with follow-up ≤ 6 months and a group with follow-up > 6 months. Compared with the control group, when the follow-up period was ≤ 6 months, the improvement in MACE incidence in the intervention group was not significant (*P* > 0.05), whereas when the follow-up period exceeded 6 months, the intervention group showed a significant reduction in MACE incidence (RR = 0.88, 95% CI: [0.79,0.97], *P* = 0.01). The forest plot are shown in Supplement Fig. 2.2.

#### Subgroup analysis based on the disease classification

Based on the different disease types of the included population, these studies were divided into an ACS group and a CCS group. Compared with the control group, the MACE incidence rate in the intervention group with CCS showed no significant improvement (*P* > 0.05), and similarly, the incidence of MACE in the intervention group with ACS also showed no significant improvement (*P* > 0.05). The forest plot are shown in Supplement Fig. 2.3.

### Secondary outcome

The secondary outcomes of this study included the incidence of, incidence of cardiac arrest (CA), incidence of gastrointestinal adverse reaction, incidence of death from any cause, incidence of revascularization, incidence of infection, hs-CRP, left ventricular ejection fraction (LVEF), IL-6, infarct size, and neutrophil count. The specific details are provided in the Table 2.

**Table 2:** Analysis of primary and secondary outcome

Outcomes	study numbers	*P* for Q test	I²	Effect mode	RR/MD(95%)	*P* for Z test
MACE	15	0.002	60%	RE	0.93 [0.85, 1.02]	0.13
All-cause mortality	13	0.67	0%	RE	0.97 [0.92, 1.03]	0.30
Cardiac arrest	3	0.93	0%	FE	0.87 [0.38, 2.01]	0.75
Revascularization	9	<0.0001	75%	RE	0.85 [0.73, 0.98]	0.03
Infection	7	0.55	0%	FE	1.06 [0.92, 1.22]	0.45
Angina	10	0.004	59%	RE	0.72 [0.58, 0.90]	0.004
Gastrointestinal adverse reaction	4	0.01	69%	RE	1.30 [0.88, 1.93]	0.19
LVEF	2	0.88	0%	FE	1.41 [0.08, 2.75]	0.04
Hs-CRP	9	<0.0001	93%	RE	−1.05 [−2.10, 0.00]	0.05
IL-6	3	<0.0001	97%	RE	−4.11 [−7.13, −1.09]	0.008
Neutrophil count	2	0.80	0%	FE	−0.74 [−1.19, −0.29]	0.001

#### Incidence of CA

Three studies [7, 26, 32] reported the cardiac arrest. The comparison of the cardiac arrest indicator between the intervention group and placebo group showed no statistically significant difference (RR = 0.87, 95% CI: [0.38, 2.01], *P* = 0.75). These results show that new immunomodulatory drugs could not reduce the incidence of CA compared with different placebo groups. The forest plot are shown in Supplement Fig. 3.

#### Incidence of angina

Ten studies [6, 7, 14, 17, 20, 26, 28, 32, 34, 36] (13 trials) reported angina incidence. The results showed that compared with the placebo group, the incidence of angina in the intervention group were markedly improved, with a statistically significant difference (RR = 0.73, 95% CI: [0.58,0.92], *P* = 0.007). These results show that new immunomodulatory drugs could reduce the incidence of angina compared with different placebo groups. The forest plot are shown in Supplement Fig. 4.

#### Incidence of all-cause mortality

Thirteen studies [6–9, 14, 17, 18, 20, 27–29, 33, 34] (16 trials) reported the incidence of death from any cause. The results showed no statistically significant difference in the death from any cause outcome between the intervention group and placebo group (RR = 0.98, 95% CI: [0.92, 1.04], *P* = 0.50). These results show that new immunomodulatory drugs could not reduce the incidence of all-cause mortality compared with different placebo groups. The forest plot are shown in Supplement Fig. 5.

#### Incidence of revascularization

Nine studies [6, 8, 9, 14, 17, 29, 30, 33, 34] (11 trials) reported the revascularization indicators. The outcomes demonstrated that the incidence of revascularization in the intervention group reduced with a statistically significant difference (RR = 0.86, 95% CI: [0.74,0.99], *P* = 0.04) compared with the different control group. These results show that new immunomodulatory drugs could reduce the incidence of revascularization compared with different placebo groups. The forest plot are shown in Supplement Fig. 6.

#### Incidence of gastrointestinal adverse effect

Four studies [7, 32, 33, 36] (5 trials) reported the incidence of gastrointestinal adverse effect. The incidence of gastrointestinal adverse showed no statistically significant difference between the intervention group and placebo group (RR = 1.22, 95% CI: [0.85, 1.74], *P* = 0.27). These results show that new immunomodulatory drugs could not reduce the incidence of revascularization compared with different placebo groups. The forest plot are shown in Supplement Fig. 7.

#### Incidence of infection

Seven studies [6–9, 18, 27, 29] encompassing 10 trials reported the incidence of infection. There was no statistically significant difference between the intervention and placebo groups (RR = 1.06, 95% CI: 0.92–1.22, *P* = 0.45). These findings indicate that the new immunomodulatory drugs did not reduce the risk of infection compared with various placebo controls. The corresponding forest plot is presented in Supplementary Fig. 12.

#### Inflammatory markers

Nine [19, 21–24, 26, 27, 35, 36] studies (10 trials) reported the hs-CRP. The results showed that there was no significant difference of hs-CRP between placebo group and intervention group (MD=−1.05, 95% CI: [−2.10,0.00], *P* = 0.05). The forest plot are shown in Supplementary Fig. 8. A total of 3 studies [25, 27, 36] (5 trials) reported IL-6. The results showed that IL-6 was markedly reduced in intervention group when compared with the different placebo groups, (MD=−4.11, 95% CI: [−7.13, −1.09, *P* = 0.008). The forest plot are shown in Supplementary Fig. 9. A total of 2 studies [22, 36] (3 trials) reported neutrophil count. The results showed that the neutrophil count in the intervention group were markedly reduced compared with the different placebo groups, (MD=−0.74, 95% CI: [−1.19, −0.29], *P* = 0.001). The forest plot are shown in Supplementary Fig. 10. These results show that compared with different placebo groups, the new immunomodulatory drugs could not reduce level of the hs-CRP, but could reduce the level of IL-6 and neutrophil count.

#### LVEF

Two studies [26, 36] (3 trials) reported data on left ventricular ejection fraction. The comparison of LVEF between the intervention group and different placebo groups showed no statistically significant difference (MD = 1.41, 95% CI: [0.08,2.75], *P* = 0.04). These results show that new immunomodulatory drugs were associated with an increase in LVEF compared with various placebo groups. The forest plot are shown in Supplementary Fig. 11.

### Publication bias, meta-regression, and sensitivity analysis

**Fig. 4:**
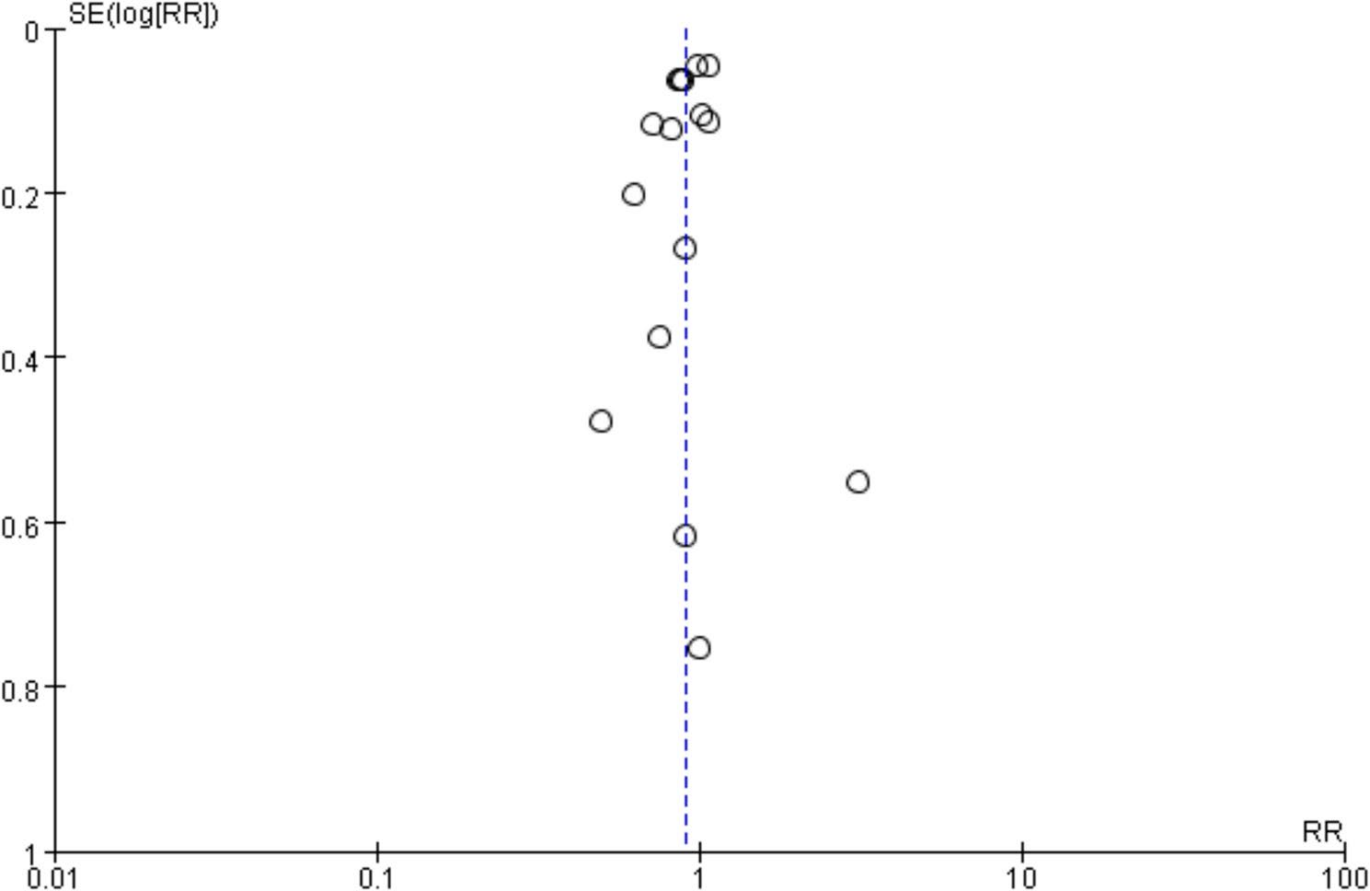
Funnel plot of MACE

The funnel plots are shown in Fig. 4, the results showed that the funnel plot was symmetrical, suggesting the absence of publication bias. In addition, both Egger’s test (*P* = 1.00) and Begg’s test (*P* = 0.84) indicated *P* > 0.05, further supporting the conclusion that publication bias is unlikely.

Meta-regression indicated that type of drugs is a significant source of heterogeneity, *P* = 0.002. The country class and year are not a significant source of heterogeneity, *P*>0.05.

In the sensitivity analysis, the point estimate for MACE remained stable (RR range: 0.91–0.95) regardless of which individual study was omitted, indicating the overall result was robust. Details information is shown in Table 3. The exclusion of certain large trials, such as Nicholls 2014 or Nidorf 2020, led to minor fluctuations in the pooled risk ratio and its confidence intervals, but these changes were not substantial enough to alter the primary conclusion of the analysis. For instance, upon excluding Nicholls 2014, the result became statistically significant (RR = 0.90, 95% CI: 0.82, 0.98, *P* = 0.02), while excluding Nidorf 2020 yielded an RR of 0.94 (95% CI: 0.86,1.03, *P* = 0.20). The fact that no single study dramatically changed the effect estimate suggests that the observed heterogeneity (I² = 60%) is not driven solely by any one trial but is more likely attributable to broader differences across studies, such as variations in drug mechanisms of action, as identified in our subgroup analysis and meta-regression.

**Table 3:** Influence plot of MACE

Incidence of MACE				
Excluded Study ID	RR(95%CI)	P for z test	I²	P for Q test
None	0.92 [0.84, 1.01]	0.0007	60%	0.09
Abbate 2020 [18]	0.92 [0.84, 1.01]	0.0004	63%	0.09
Akrami 2021 [20]	0.93 [0.85, 1.02]	0.11	61%	0.0008
MEWTON 2021 [26]	0.92 [0.84, 1.01]	0.10	63%	0.0004
Morton 2015 [27]	0.92 [0.84, 1.00]	0.05	58%	0.002
Nicholls 2014 [28]	0.90 [0.82, 0.98]	0.02	54%	0.006
Nidorf 2020 [8]	0.94 [0.86, 1.03]	0.20	56%	0.0003
O’Donoghue 2014 [29]	0.91 [0.82, 1.02]	0.11	62%	0.0005
O’Donoghue 2016 [30]	0.91 [0.83, 1.01]	0.07	61%	0.0006
Ridker 2017(a) [6]	0.93 [0.84, 1.03]	0.16	61%	0.0008
Ridker 2017(b) [6]	0.93 [0.85, 1.03]	0.17	60%	0.001
Ridker 2017(c) [6]	0.93 [0.84, 1.03]	0.16	60%	0.0010
Ridker 2019 [9]	0.91 [0.83, 1.01]	0.08	62%	0.0005
Roubille 2024 [32]	0.94 [0.86, 1.03]	0.17	58%	0.002
Shah 2020 [33]	0.92 [0.84, 1.02]	0.10	62%	0.0004
Tardif 2019 [7]	0.93 [0.85, 1.03]	0.15	61%	0.0008
Tong 2020 [34]	0.93 [0.84, 1.02]	0.11	62%	0.0005
WHITE 2014 [14]	0.90 [0.82, 1.00]	0.05	55%	0.004

### Quality of the evidence

This study conducted GRADE evidence ratings for the primary outcome and secondary outcomes. The evidence quality for the incidence of MACEs was accessed as moderate. This indicates that the current evidence is highly credible regarding the efficacy and safety of new immunomodulatory drugs therapy in patients with CHD. Detailed results are presented in Table 4.

**Table 4:** Quality of the evidence (GRADE)

Outcome Measure	No. of Studies	Risk of Bias	Inconsistency	Indirectness	Imprecision	Publication Bias	GRADE Rating
MACE	15	Not downgraded	Downgrade b	Not downgraded	Not downgraded	Not downgraded	Moderate
Death from any cause	16	Not downgraded	Not downgraded	Not downgraded	Not downgraded	Not downgraded	Moderate
Cardiac arrest	3	Not downgraded	Not downgraded	Not downgraded	Not downgraded	Not downgraded	High
Angina	10	Not downgraded	Downgrade b	Not downgraded	Not downgraded	Not downgraded	Low
Gastrointestinal adverse reaction	4	Not downgraded	Downgrade b	Not downgraded	Not downgraded	Not downgraded	Low
Revascularization	9	Not downgraded	Downgrade b	Not downgraded	Not downgraded	Not downgraded	Low
Hs-CRP	9	Not downgraded	Downgrade b	Not downgraded	Not downgraded	Downgrade c	Low
Left ventricular ejection fraction	2	Not downgraded	Not downgraded	Not downgraded	Not downgraded	Not downgraded	High
IL-6	3	Not downgraded	Downgrade b	Not downgraded	Not downgraded	Not downgraded	Moderate
Neutrophil count	2	Not downgraded	Not downgraded	Not downgraded	Not downgraded	Not downgraded	High

## Discussion

Our meta-analysis systematically reviewed the efficacy and safety of new immunomodulatory drugs for reducing MACE in patients with CHD, as well as related clinical indicators such as incidence of revascularization and angina. It also conducted a comprehensive analysis of the new immunomodulatory drug’s application on cytokines like hs-CRP and IL-6. This study synthesized evidence from 25 RCTs. The evidence indicates while the overall effect on MACE was not significant in CHD patients, the new immunomodulatory drugs could reduced incidence of angina, revascularization, IL-6 levels, neutrophil count levels, and improve the LVEF.

The overall analysis indicated that adding immunomodulatory drugs to standard therapy did not significantly reduce MACE incidence (RR = 0.92, 95% CI: [0.84, 1.01], *P* = 0.09). However, this null finding masks substantial heterogeneity. Subgroup analysis by drug class demonstrated NLRP3 inflammasome inhibitor (RR = 0.75, 95% CI: [0.65,0.86], *P* < 0.0001) and IL pathway inhibitors (RR = 0.88, 95% CI: [0.80,0.97], *P* = 0.009) significantly reduced MACE, whereas broad-spectrum immunomodulator showed no benefit. These dichotomous data suggested that effect may changed with different anti-inflammatory pathway. Furthermore, a treatment duration exceeding six months was necessary to observe a significant MACE reduction (RR = 0.89,95%CI: [0.82,0.98], *P* = 0.01). indicating that sustained immunomodulation is required for clinical benefit. In contrast, disease classifications (ACS vs. CCS) did not affect outcomes, which demonstrated the new immunomodulatory drugs have no benefits for all sorts of CHD patients.

The significant reduction in MACE with colchicine (RR = 0.75, 95% CI: [0.65, 0.86],*P* < 0.0001) are consistent with the COLCOT and LoDoCo2 trials, Colchicine is thought to stabilize atherosclerotic plaques by inhibiting NLRP3 inflammasome mediated production and neutrophil activity [8, 32]. By inhibiting neutrophil chemotaxis, adhesion, and activation of the NLRP3 inflammasome, colchicine can reduce the production of interleukin (IL)−1β and IL-18 [8]. Similarly, the clinical benefit of canakinumab (RR = 0.87, 95% CI: [0.81, 0.94], *P* = 0.0002) aligns with the CANTOS trial, confirming that blocking this upstream cytokine reduces vascular inflammation independent of lipid regulation [6]. These findings further support the mechanistic theory that targeting upstream inflammatory mediators (e.g., IL-1β, NLRP3) can block the pro-inflammatory cascades driving atherosclerosis. Conversely, the lack of benefit with methotrexate, Lp-PLA2 inhibitors, and p38 MAPK inhibitors is consistent with previous negative trials such as CIRT and SOLID-TIMI 52 [14, 28]. As broad or downstream anti-inflammatory strategies may fail to modulate key pathways of plaque destabilization. The other reason why the broad-spectrum immunomodulator, Lp-PLA2 inhibitor, and p38 MAPK kinase inhibitor showed no benefits in CHD patients is that the involved patients are not enough in quantity. It reminds us that require more RCTs about these drugs in CHD patients to provide sufficient statistics.

We suppose the efficacy of a drug is profoundly influenced by its molecular target within the complex cascade of coronary inflammation. We surprisingly found the effective drugs are targeting upstream, central inflammatory pathways, the upstream inhibition effectively dampens the entire subsequent inflammatory cascade (e.g., reduced IL-1β, IL-6, neutrophil count), leading to plaque stabilization and a significant reduction in MACE, as consistently shown in COLCOT and LoDoCo2 trials cited in our manuscript. However the ineffective drugs are targeting downstream or alternative pathways. The inflammatory cascade, once initiated upstream by NLRP3/IL-1β, may bypass the downstream pathway, or Lp-PLA2 inhibition may simply be insufficient to quell the established inflammatory response driving clinical events, and for p38 MAPK kinase inhibitors and broad-spectrum immunomodulators, their inhibition may be too broad yet not precise enough, or may be compensated by alternative pathways.

In our analysis, therapy of short term showed no benefit in reducing MACE (follow-up ≤ 6 months) may reflect delayed inhibition of neutrophil driven reperfusion injury, as indicated by the conflicting infarct size outcomes reported by Deftereos et al. and Mewton et al. [22, 26]. Notably, in Mewton’s trial, the association of colchicine with an increased risk of left ventricular thrombosis highlights its complex interaction with platelet activation and endothelial repair mechanisms, potentially mediated by microtubule-dependent thrombospondin-1 release [37].

This study still has several limitations. Firstly, the number of studies sample is not enough for Lp-PLA2 Inhibitors, p38 MAPK inhibitors, broad-spectrum immunomodulators, which limits the persuasiveness of subgroup comparisons; the lack of significant differences in reduction of MACE based on disease classification (acute vs. chronic coronary heart disease) may reflect insufficient statistical power due to small subgroup sample sizes rather than true therapeutic equivalence. Secondly, although including the latest trials could provide the latest data, excluding pre-2014 studies may omit historically relevant data. Thirdly, key prognostic variables for coronary artery disease are missing, left ventricular ejection fraction (LVEF) is a critical factor, yet only three studies report it. This degree of missingness is a major limitation that could materially affect the conclusions. Fourthly, some of the included original studies did not directly report the number of patients experiencing MACE—as predefined by us as a composite of cardiovascular death, non-fatal myocardial infarction, and non-fatal stroke—but instead reported the incidence of each component separately. when a study did not report the composite endpoint at all—but did provide separate, complete data for all three component events—did we sum the components to estimate the total number of MACE events. our method could overestimate the true number of patients with MACE.

This meta-analysis demonstrates that despite efficacy in different drugs and follow-up durations is not the same, some specific new immunomodulatory drugs but not all new immunomodulator can provide significant cardiovascular protection for patients with CHD. The other type of drugs do not have benefits may contribute to insufficient sample size. This result showed that we should carry out more researches to identify the effects of broad-spectrum immunomodulators, PhospholipaseA2 inhibitors and P38MAPK kinase inhibitors in CHD patients. Our meta analysis supports the concept of precise anti immunomodulatory therapy in CHD patients. Rather than applying a blanket “anti immunomodulatory” strategy, clinicians should prioritize drugs with proven efficacy and mechanistic rationale, specifically NLRP3 inflammasome inhibitors (e.g., colchicine) and IL-1β pathway inhibitors (e.g., canakinumab) should be considered for secondary prevention in CHD patients. Conversely, based on current evidence, broad-spectrum immunomodulators (e.g., methotrexate) and downstream inhibitors (e.g., Lp-PLA2 inhibitors, p38 MAPK inhibitors) should not be used in CHD patients, as they have not demonstrated significant benefit.

This meta-analysis provides evidence that supports the selective use of colchicine and IL-1 inhibitors in the secondary prevention of CHD, particularly in patients with high inflammatory burden and when used long-term. It is anticipated that future updates to ESC and AHA guidelines may strengthen recommendations for colchicine in both ACS and chronic coronary syndromes and provide more accurate guidance on IL-1 inhibition, potentially reserved for specific high-risk subgroups.

These findings reinforce the theory of inflammation as a modifiable cardiovascular risk factor and pave the way for precision medicine in atherosclerosis management.

## Supplementary Information

Supplementary Material 1.

## Data Availability

The original contributions presented in the study are included in the article/supplementary material, and further inquiries can be directed to the corresponding author.

## References

[1] TSAO C W, ADAY A W, ALMARZOOQ Z I, et al. Heart disease and stroke Statistics-2023 update: A report from the American heart association [J]. Circulation. 2023;147(8):e93–621. 36695182 10.1161/CIR.0000000000001123 PMC12135016

[2] KOVACH C P, HEBBE A, GLORIOSO T J, et al. Association of residual ischemic disease with clinical outcomes after percutaneous coronary intervention [J]. JACC Cardiovasc Interv. 2022;15(24):2475–86. 36543441 10.1016/j.jcin.2022.11.002

[3] VISSEREN F L J, MACH F, SMULDERS Y M, et al. 2021 ESC guidelines on cardiovascular disease prevention in clinical practice [J]. Eur J Prev Cardiol. 2022;29(1):5–115. 34558602 10.1093/eurjpc/zwab154

[4] KARAKAYALI M, ALTUNOVA M, YAKISAN T, et al. The relationship between the systemic Immune-Inflammation index and ischemia with Non-Obstructive coronary arteries in patients undergoing coronary angiography [J]. Arq Bras Cardiol. 2024;121(2):e20230540. 38597536 10.36660/abc.20230540 PMC12092018

[5] Karakayali M, Ogun M, Artac I, et al. Serum malondialdehyde levels at admission as a predictor of inhospital mortality in patients with acute coronary syndrome. Coron Artery Dis. 2025;36(3):211–7. 39620872 10.1097/MCA.0000000000001469

[6] Ridker PM, Everett BM, Thuren T, et al. Antiinflammatory therapy with canakinumab for atherosclerotic disease. N Engl J Med. 2017;377(12):1119–31. 28845751 10.1056/NEJMoa1707914

[7] TARDIF JC, WATERS D D KOUZS, et al. Efficacy and safety of Low-Dose Colchicine after myocardial infarction [J]. N Engl J Med. 2019;381(26):2497–505. 31733140 10.1056/NEJMoa1912388

[8] Nidorf SM, Fiolet ATL, Mosterd A, et al. Colchicine in patients with chronic coronary disease. N Engl J Med. 2020;383(19):1838–47. 32865380 10.1056/NEJMoa2021372

[9] Ridker PM, Everett BM, Pradhan A, et al. Low-dose methotrexate for the prevention of atherosclerotic events. N Engl J Med. 2019;380(8):752–62. 30415610 10.1056/NEJMoa1809798 PMC6587584

[10] Younas A, Awan Z, Khan T, et al. The effect of Colchicine on myocardial infarction: an updated systematic review and meta-analysis of randomized controlled trials. Curr Probl Cardiol. 2025;50(1):102878. 39393620 10.1016/j.cpcardiol.2024.102878

[11] KNUUTI J, WIJNS W. 2019 ESC guidelines for the diagnosis and management of chronic coronary syndromes [J]. Eur Heart J. 2020;41(3):407–77. 31504439 10.1093/eurheartj/ehz425

[12] VIRANI SS, NEWBY L K, ARNOLD SV et al. AHA/ACC/ACCP/ASPC/NLA/PCNA guideline for the management of patients with chronic coronary disease: a report of the American. 2023

[13] Sterne JA, Sutton AJ, Ioannidis JP, et al. Recommendations for examining and interpreting funnel plot asymmetry in meta-analyses of randomised controlled trials [J]. BMJ. 2011;343:d4002. 21784880 10.1136/bmj.d4002

[14] WHITE HD, HELD C, STEWART R, et al. Darapladib for preventing ischemic events in stable coronary heart disease [J]. N Engl J Med. 2014;370(18):1702–11. 24678955 10.1056/NEJMoa1315878

[15] BEGG C B MAZUMDARM. Operating characteristics of a rank correlation test for publication bias [J]. Biometrics. 1994;50(4):1088–101. 7786990

[16] Egger M, Davey Smith G, Schneider M, et al. Bias in meta-analysis detected by a simple, graphical test. BMJ. 1997;315(7109):629–34. 9310563 10.1136/bmj.315.7109.629 PMC2127453

[17] Abbate A, Kontos MC, Abouzaki NA, et al. Comparative safety of interleukin-1 blockade with Anakinra in patients with ST-segment elevation acute myocardial infarction (from the VCU-ART and VCU-ART2 pilot studies). Am J Cardiol. 2015;115(3):288–92. 25482680 10.1016/j.amjcard.2014.11.003

[18] TRANKLE C R ABBATEA, BUCKLEY L F, et al. Interleukin-1 Blockade inhibits the acute inflammatory response in patients with ST-Segment-Elevation myocardial infarction [J]. J Am Heart Assoc. 2020;9(5):e014941. 32122219 10.1161/JAHA.119.014941 PMC7335541

[19] Akodad M, Lattuca B, Nagot N, et al. COLIN trial: value of colchicine in the treatment of patients with acute myocardial infarction and inflammatory response. Arch Cardiovasc Dis. 2017;110(6–7):395–402. 28065445 10.1016/j.acvd.2016.10.004

[20] Akrami M, Izadpanah P, Bazrafshan M, et al. Effects of colchicine on major adverse cardiac events in next 6-month period after acute coronary syndrome occurrence; a randomized placebo-control trial. BMC Cardiovasc Disord. 2021;21(1):583. 34876021 10.1186/s12872-021-02393-9 PMC8650300

[21] Broch K, Anstensrud AK, Woxholt S, et al. Randomized trial of interleukin-6 receptor inhibition in patients with acute ST-segment elevation myocardial infarction. J Am Coll Cardiol. 2021;77(15):1845–55. 33858620 10.1016/j.jacc.2021.02.049

[22] Deftereos S, Giannopoulos G, Angelidis C, et al. Anti-inflammatory treatment with colchicine in acute myocardial infarction: a pilot study. Circulation. 2015;132(15):1395–403. 26265659 10.1161/CIRCULATIONAHA.115.017611

[23] Hennessy T, Soh L, Bowman M, et al. The low dose Colchicine after myocardial infarction (LoDoCo-MI) study: a pilot randomized placebo controlled trial of Colchicine following acute myocardial infarction. Am Heart J. 2019;215:62–9. 31284074 10.1016/j.ahj.2019.06.003

[24] Kajikawa M, Higashi Y, Tomiyama H, et al. Effect of short-term Colchicine treatment on endothelial function in patients with coronary artery disease. Int J Cardiol. 2019;281:35–9. 30683457 10.1016/j.ijcard.2019.01.054

[25] MARTíNEZ GJ, Robertson S, Barraclough J, et al. Colchicine acutely suppresses local cardiac production of inflammatory cytokines in patients with an acute coronary syndrome. J Am Heart Assoc. 2015;4(8):e002128. 26304941 10.1161/JAHA.115.002128 PMC4599469

[26] MEWTON N, ROUBILLE F, BRESSON D, et al. Effect of Colchicine on myocardial injury in acute myocardial infarction [J]. Circulation. 2021;144(11):859–69. 34420373 10.1161/CIRCULATIONAHA.121.056177 PMC8462445

[27] Morton AC, Rothman AM, Greenwood JP, et al. The effect of interleukin-1 receptor antagonist therapy on markers of inflammation in non-ST elevation acute coronary syndromes: the MRC-ILA heart study. Eur Heart J. 2015;36(6):377–84. 25079365 10.1093/eurheartj/ehu272 PMC4320321

[28] Nicholls SJ, Kastelein JJ, Schwartz GG, et al. Varespladib and cardiovascular events in patients with an acute coronary syndrome: the VISTA-16 randomized clinical trial. JAMA. 2014;311(3):252–62. 24247616 10.1001/jama.2013.282836

[29] O’DONOGHUE M L BRAUNWALDE, WHITE H D, et al. Effect of Darapladib on major coronary events after an acute coronary syndrome: the SOLID-TIMI 52 randomized clinical trial [J]. JAMA. 2014;312(10):1006–15. 25173516 10.1001/jama.2014.11061

[30] O’Donoghue ML, Glaser R, Cavender MA, et al. Effect of losmapimod on cardiovascular outcomes in patients hospitalized with acute myocardial infarction: a randomized clinical trial. JAMA. 2016;315(15):1591–9. 27043082 10.1001/jama.2016.3609

[31] Psaltis PJ, Nguyen MT, Singh K, et al. Optical coherence tomography assessment of the impact of Colchicine on non-culprit coronary plaque composition after myocardial infarction. Cardiovasc Res. 2024;121(3):468–78. 39189611 10.1093/cvr/cvae191

[32] Roubille F, Bouabdallaoui N. Low-dose colchicine in patients with type 2 diabetes and recent myocardial infarction in the colchicine cardiovascular outcomes trial (COLCOT). Diabetes Care. 2024;47(3):467–70. 38181203 10.2337/dc23-1825

[33] Shah B, Pillinger M, Zhong H, et al. Effects of acute Colchicine administration prior to percutaneous coronary intervention: COLCHICINE-PCI randomized trial. Circ Cardiovasc Interv. 2020;13(4):e008717. 32295417 10.1161/CIRCINTERVENTIONS.119.008717 PMC7169992

[34] Tong DC, Quinn S, Nasis A, et al. Colchicine in patients with acute coronary syndrome: the Australian COPS randomized clinical trial. Circulation. 2020;142(20):1890–900. 32862667 10.1161/CIRCULATIONAHA.120.050771

[35] VAIDYA K, ARNOTT C, MARTíNEZ GJ, et al. Colchicine therapy and plaque stabilization in patients with acute coronary syndrome: A CT coronary angiography study [J]. JACC Cardiovasc Imaging. 2018;11(2 Pt 2):305–16. 29055633 10.1016/j.jcmg.2017.08.013

[36] XU X, XIAO J. Colchicine in acute coronary syndromes: efficacy and safety assessment [J]. Chin Adm Health Stand. 2023;14(23):139–42.

[37] CORBAN M T, LERMAN L O LERMANA. Endothelial dysfunction [J]. Arterioscler Thromb Vasc Biol. 2019;39(7):1272–4. 31242027 10.1161/ATVBAHA.119.312836

