# Supplementary material for: Efficacy of new immunomodulatory drugs on major adverse cardiovascular events in patients with coronary heart disease: a systematic review and meta-analysis of randomized controlled trials
Source: BMC Cardiovasc Disord. 2025 Nov 10;25:798. doi: 10.1186/s12872-025-05250-1 (PMC12604377; doi:10.1186/s12872-025-05250-1)
Supplement: Supplementary file 1 — Supplementary Material 1. [file 12872_2025_5250_MOESM1_ESM.docx]

Table1

Web of science 132+ embase 180+ pubmed 7640+ cochrane 607 +Wangfang 619 + CNKI 47+VIP 18+sinomed 84=9329.

**Web of science**

| Search | Query | Results |
| --- | --- | --- |
| #5 | #3 AND #4  ((((TS=(coronary artery disease)) OR TS=(coronary disease)) OR TS=(ischemic heart disease)) AND #7) AND TS=("Randomized controlled trial"[Publication Type] OR "controlled clinical trial"[Publication Type] OR "randomized"[Title/Abstract] OR "placebo"[Title/Abstract] OR "drug therapy"[MeSH Subheading] OR "randomly"[Title/Abstract] OR "trial"[Title/Abstract] OR "groups"[Title/Abstract]) | 132 |
| #4 | TS=("Randomized controlled trial"[Publication Type] OR "controlled clinical trial"[Publication Type] OR "randomized"[Title/Abstract] OR "placebo"[Title/Abstract] OR "drug therapy"[MeSH Subheading] OR "randomly"[Title/Abstract] OR "trial"[Title/Abstract] OR "groups"[Title/Abstract]) | 4564 |
| #3 | #1 AND #2 | 1057 |
| #2 | (((TS=(Anti-Inflammatory Agents)) OR TS=(Interleukins)) OR TS=(colchicine)) OR TS=(methotrexate) | 79，795 |
| #1 | (TS=(coronary disease)) OR TS=(coronary heart disease) | 184，660 |

**Pubmed(Public medicine)**

| Search | Query | Results |
| --- | --- | --- |
| #5 | #3 AND #4  ("anti inflammatory agents"[MeSH Terms] OR ("anti inflammatory agents"[Pharmacological Action] OR "anti inflammatory agents"[MeSH Terms] OR ("anti inflammatory"[All Fields] AND "agents"[All Fields]) OR "anti inflammatory agents"[All Fields] OR ("anti"[All Fields] AND "inflammatory"[All Fields] AND "agents"[All Fields]) OR "anti inflammatory agents"[All Fields]) OR "interleukins"[MeSH Terms] OR ("interleukine"[All Fields] OR "interleukines"[All Fields] OR "interleukins"[MeSH Terms] OR "interleukins"[All Fields] OR "interleukin"[All Fields]) OR "colchicine"[MeSH Terms] OR ("colchicine"[MeSH Terms] OR "colchicine"[All Fields] OR "colchicin"[All Fields] OR "colchicines"[All Fields] OR "colchicine s"[All Fields]) OR "methotrexate"[MeSH Terms] OR ("methotrexate"[MeSH Terms] OR "methotrexate"[All Fields] OR "methotrexate s"[All Fields] OR "methotrexates"[All Fields])) AND ("coronary disease"[MeSH Terms] OR ("coronary disease"[MeSH Terms] OR ("coronary"[All Fields] AND "disease"[All Fields]) OR "coronary disease"[All Fields]) OR ("coronary disease"[MeSH Terms] OR ("coronary"[All Fields] AND "disease"[All Fields]) OR "coronary disease"[All Fields] OR ("coronary"[All Fields] AND "heart"[All Fields] AND "disease"[All Fields]) OR "coronary heart disease"[All Fields]) OR "coronary artery disease"[MeSH Terms] OR ("coronary artery disease"[MeSH Terms] OR ("coronary"[All Fields] AND "artery"[All Fields] AND "disease"[All Fields]) OR "coronary artery disease"[All Fields])) AND ("Randomized controlled trial"[Publication Type] OR "controlled clinical trial"[Publication Type] OR "randomized"[Title/Abstract] OR "placebo"[Title/Abstract] OR "drug therapy"[MeSH Subheading] OR "randomly"[Title/Abstract] OR "trial"[Title/Abstract] OR "groups"[Title/Abstract]) | 7640 |
| #4 | "Randomized controlled trial"[Publication Type] OR "controlled clinical trial"[Publication Type] OR "randomized"[Title/Abstract] OR "placebo"[Title/Abstract] OR "drug therapy"[MeSH Subheading] OR "randomly"[Title/Abstract] OR "trial"[Title/Abstract] OR "groups"[Title/Abstract] | 6,392,121 |
| #3 | #1 AND #2  ("anti inflammatory agents"[MeSH Terms] OR ("anti inflammatory agents"[Pharmacological Action] OR "anti inflammatory agents"[MeSH Terms] OR ("anti inflammatory"[All Fields] AND "agents"[All Fields]) OR "anti inflammatory agents"[All Fields] OR ("anti"[All Fields] AND "inflammatory"[All Fields] AND "agents"[All Fields]) OR "anti inflammatory agents"[All Fields]) OR "interleukins"[MeSH Terms] OR ("interleukine"[All Fields] OR "interleukines"[All Fields] OR "interleukins"[MeSH Terms] OR "interleukins"[All Fields] OR "interleukin"[All Fields]) OR "colchicine"[MeSH Terms] OR ("colchicine"[MeSH Terms] OR "colchicine"[All Fields] OR "colchicin"[All Fields] OR "colchicines"[All Fields] OR "colchicine s"[All Fields]) OR "methotrexate"[MeSH Terms] OR ("methotrexate"[MeSH Terms] OR "methotrexate"[All Fields] OR "methotrexate s"[All Fields] OR "methotrexates"[All Fields])) AND ("coronary disease"[MeSH Terms] OR ("coronary disease"[MeSH Terms] OR ("coronary"[All Fields] AND "disease"[All Fields]) OR "coronary disease"[All Fields]) OR ("coronary disease"[MeSH Terms] OR ("coronary"[All Fields] AND "disease"[All Fields]) OR "coronary disease"[All Fields] OR ("coronary"[All Fields] AND "heart"[All Fields] AND "disease"[All Fields]) OR "coronary heart disease"[All Fields]) OR "coronary artery disease"[MeSH Terms] OR ("coronary artery disease"[MeSH Terms] OR ("coronary"[All Fields] AND "artery"[All Fields] AND "disease"[All Fields]) OR "coronary artery disease"[All Fields])) | 13,431 |
| #2 | "anti inflammatory agents"[MeSH Terms] OR ("anti inflammatory agents"[Pharmacological Action] OR "anti inflammatory agents"[MeSH Terms] OR ("anti inflammatory"[All Fields] AND "agents"[All Fields]) OR "anti inflammatory agents"[All Fields] OR ("anti"[All Fields] AND "inflammatory"[All Fields] AND "agents"[All Fields]) OR "anti inflammatory agents"[All Fields]) OR "interleukins"[MeSH Terms] OR ("interleukine"[All Fields] OR "interleukines"[All Fields] OR "interleukins"[MeSH Terms] OR "interleukins"[All Fields] OR "interleukin"[All Fields]) OR "colchicine"[MeSH Terms] OR ("colchicine"[MeSH Terms] OR "colchicine"[All Fields] OR "colchicin"[All Fields] OR "colchicines"[All Fields] OR "colchicine s"[All Fields]) OR "methotrexate"[MeSH Terms] OR ("methotrexate"[MeSH Terms] OR "methotrexate"[All Fields] OR "methotrexate s"[All Fields] OR "methotrexates"[All Fields]) | 1,137,946 |
| #1 | "coronary disease"[MeSH Terms] OR ("coronary disease"[MeSH Terms] OR ("coronary"[All Fields] AND "disease"[All Fields]) OR "coronary disease"[All Fields]) OR ("coronary disease"[MeSH Terms] OR ("coronary"[All Fields] AND "disease"[All Fields]) OR "coronary disease"[All Fields] OR ("coronary"[All Fields] AND "heart"[All Fields] AND "disease"[All Fields]) OR "coronary heart disease"[All Fields]) OR "coronary artery disease"[MeSH Terms] OR ("coronary artery disease"[MeSH Terms] OR ("coronary"[All Fields] AND "artery"[All Fields] AND "disease"[All Fields]) OR "coronary artery disease"[All Fields]) | 383,851 |

**Cochrane library**

| Search | Query | Results |
| --- | --- | --- |
| #5 | #3 AND #4 | 607 |
| #4 | (Randomized controlled trial):ti,ab,kw | 22452 |
| #3 | #1 AND #2 | 1163 |
| #2 | (Anti-Inflammatory Agents):ti,ab,kw OR (Interleukins):ti,ab,kw OR (Colchicine):ti,ab,kw OR (Methotrexate):ti,ab,kw | 57196 |
| #1 | ("coronary disease"):ti,ab,kw OR ("coronary heart disease"):ti,ab,kw OR (coronary artery disease):ti,ab,kw | 39060 |

**Embase(Excerpta medica database)**

| Search | Query | Results |
| --- | --- | --- |
| #5 | #3 AND #4 | 180 |
| #4 | (Randomized controlled trial):ti,ab,kw | 343,458 |
| #3 | #1 AND #2 | 1435 |
| #2 | (Anti-Inflammatory Agents):ti,ab,kw OR (Interleukins):ti,ab,kw OR (Colchicine):ti,ab,kw OR (Methotrexate):ti,ab,kw | 163，854 |
| #1 | ("coronary disease"):ti,ab,kw OR ("coronary heart disease"):ti,ab,kw OR (coronary artery disease):ti,ab,kw | 332，182 |

**VIP(Weipu information database)**

| Search | Query | Results |
| --- | --- | --- |
| #5 | #3 AND #4 | 18 |
| #4 | U=（随机对照研究） | 40，196 |
| #3 | #1 AND #2  U=（冠心病 OR 心肌梗死 OR 冠状动脉粥样硬化性心肌病 OR 急性冠状动脉综合征）AND U=（抗炎药 OR 白介素 OR 秋水仙碱 OR 甲氨蝶呤） | 2067 |
| #2 | U=（冠心病 OR 心肌梗死 OR 冠状动脉粥样硬化性心肌病 OR 急性冠状动脉综合征） | 330，146 |
| #1 | U=（抗炎药 OR 白介素 OR 秋水仙碱 OR 甲氨蝶呤） | 83，181 |

English translations

| Search | Query | Results |
| --- | --- | --- |
| #5 | #3 AND #4 | 18 |
| #4 | U=（RCT） | 40，196 |
| #3 | #1 AND #2  U = (coronary heart disease OR myocardial infarction OR coronary artery disease OR acute coronary syndrome) AND U = (anti-inflammatory agents OR interleukin OR colchicine OR methotrexate) | 2067 |
| #2 | U = (coronary heart disease OR myocardial infarction OR coronary artery disease OR acute coronary syndrome). | 330，146 |
| #1 | U = (anti-inflammatory agents OR interleukin OR colchicine OR methotrexate). | 83，181 |

**CNKI(China National Knowledge Infrastructure)**

| Search | Query | Results |
| --- | --- | --- |
| #5 | #3 AND #4  TKA=(抗炎药 OR 白介素 OR 秋水仙碱 OR 甲氨蝶呤) AND TKA=（冠心病 OR 心肌梗死 OR 冠状动脉粥样硬化性心肌病 OR 急性冠状动脉综合征）AND TKA=(随机对照） | 47 |
| #4 | TKA=(随机对照研究) | 55，700 |
| #3 | #1 AND #2  TKA=(抗炎药 OR 白介素 OR 秋水仙碱 OR 甲氨蝶呤) AND TKA=（冠心病 OR 心肌梗死 OR 冠状动脉粥样硬化性心肌病 OR 急性冠状动脉综合征） | 313 |
| #2 | TKA=(抗炎药 OR 白介素 OR 秋水仙碱 OR 甲氨蝶呤) | 61，300 |
| #1 | TKA=( 冠心病 OR 心肌梗死 OR 急性冠状动脉综合征) | 341，400 |

English translations

| Search | Query | Results |
| --- | --- | --- |
| #5 | #3 AND #4  TKA=(anti-inflammatory agents OR interleukin OR colchicine OR methotrexate) AND TKA=(coronary heart disease OR myocardial infarction OR coronary artery disease OR acute coronary syndrome) AND TKA=(randomized controlled trial). | 47 |
| #4 | TKA=(RCT) | 55，700 |
| #3 | #1 AND #2  TKA=(anti-inflammatory agents OR interleukin OR colchicine OR methotrexate) AND TKA=(coronary heart disease OR myocardial infarction OR coronary artery disease OR acute coronary syndrome). | 313 |
| #2 | TKA=(anti-inflammatory agents OR interleukin OR colchicine OR methotrexate). | 61，300 |
| #1 | TKA=(coronary heart disease OR myocardial infarction OR acute coronary syndrome). | 341，400 |

**Wangfang (wanfang data knowledge service platform)**

| Search | Query | Results |
| --- | --- | --- |
| #5 | #3 AND #4  (主题:(抗炎药) or 主题:(白介素) or 主题:(秋水仙碱) or 主题:(甲氨蝶呤) )AND (主题:(冠心病) or 主题:(心肌梗死) or 主题:(冠状动脉粥样硬化性心肌病) or 主题:(急性冠状动脉综合征)) AND 主题:(随机对照试验) | 619 |
| #4 | 主题:(随机对照试验) | 51，377 |
| #3 | #1 AND #2  (主题:(抗炎药) or 主题:(白介素) or 主题:(秋水仙碱) or 主题:(甲氨蝶呤) )AND (主题:(冠心病) or 主题:(心肌梗死) or 主题:(冠状动脉粥样硬化性心肌病) or 主题:(急性冠状动脉综合征)) | 3,106 |
| #2 | 主题:(抗炎药) or 主题:(白介素) or 主题:(秋水仙碱) or 主题:(甲氨蝶呤) | 113，250 |
| #1 | 主题:(冠心病) or 主题:(心肌梗死) or 主题:(冠状动脉粥样硬化性心肌病) or 主题:(急性冠状动脉综合征) | 425，486 |

English translations

| Search | Query | Results |
| --- | --- | --- |
| #5 | #3 AND #4  (Subject:(anti-inflammatory agents) OR Subject:(interleukin) OR Subject:(colchicine) OR Subject:(methotrexate)) AND (Subject:(coronary heart disease) OR Subject:(myocardial infarction) OR Subject:(coronary artery disease) OR Subject:(acute coronary syndrome)) AND Subject:(randomized controlled trial). | 619 |
| #4 | Subject: (randomized controlled trial). | 51，377 |
| #3 | #1 AND #2  (Subject:(anti-inflammatory agents) OR Subject:(interleukin) OR Subject:(colchicine) OR Subject:(methotrexate)) AND (Subject:(coronary heart disease) OR Subject:(myocardial infarction) OR Subject:(coronary artery disease) OR Subject:(acute coronary syndrome)). | 3,106 |
| #2 | Subject:(anti-inflammatory agents) OR Subject:(interleukin) OR Subject:(colchicine) OR Subject:(methotrexate) | 113，250 |
| #1 | Subject:(coronary heart disease) OR Subject:(myocardial infarction) OR Subject:(coronary artery disease) OR Subject:(acute coronary syndrome) | 425，486 |

**Sinomed(China Biomedical Literature Database)**

| Search | Query | Results |
| --- | --- | --- |
| #5 | #3 AND #4  ("随机对照研究"[常用字段:智能] OR "随机对照实验"[常用字段:智能] OR "rct"[常用字段:智能]) AND ("抗炎药"[常用字段:智能] OR "白介素"[常用字段:智能] OR "秋水仙碱"[常用字段:智能] OR "甲氨蝶呤"[常用字段:智能] )AND ("冠心病"[常用字段:智能] OR "心肌梗死"[常用字段:智能] OR "冠状动脉粥样硬化性心肌病"[常用字段:智能] OR "急性冠状动脉综合征"[常用字段:智能]) | 84 |
| #4 | "随机对照研究"[常用字段:智能] OR "随机对照实验"[常用字段:智能] OR "rct"[常用字段:智能] | 31821 |
| #3 | #1 AND #2  ("抗炎药"[常用字段:智能] OR "白介素"[常用字段:智能] OR "秋水仙碱"[常用字段:智能] OR "甲氨蝶呤"[常用字段:智能] )AND ("冠心病"[常用字段:智能] OR "心肌梗死"[常用字段:智能] OR "冠状动脉粥样硬化性心肌病"[常用字段:智能] OR "急性冠状动脉综合征"[常用字段:智能]) | 2557 |
| #2 | "抗炎药"[常用字段:智能] OR "白介素"[常用字段:智能] OR "秋水仙碱"[常用字段:智能] OR "甲氨蝶呤"[常用字段:智能] | 108，990 |
| #1 | "冠心病"[常用字段:智能] OR "心肌梗死"[常用字段:智能] OR "冠状动脉粥样硬化性心肌病"[常用字段:智能] OR "急性冠状动脉综合征"[常用字段:智能] | 342，098 |

English translations

| Search | Query | Results |
| --- | --- | --- |
| #5 | #3 AND #4  ("randomized controlled trial"[All Fields] OR "randomized controlled study"[All Fields] OR "rct"[All Fields]) AND ("anti-inflammatory agents"[All Fields] OR "interleukin"[All Fields] OR "colchicine"[All Fields] OR "methotrexate"[All Fields]) AND ("coronary heart disease"[All Fields] OR "myocardial infarction"[All Fields] OR "coronary artery disease"[All Fields] OR "acute coronary syndrome"[All Fields]) | 84 |
| #4 | "randomized controlled trial"[Common Fields: Intelligent] OR "randomized controlled study"[Common Fields: Intelligent] OR "rct"[Common Fields: Intelligent] | 31821 |
| #3 | #1 AND #2  ("anti-inflammatory agents"[Common Fields: Intelligent] OR "interleukin"[Common Fields: Intelligent] OR "colchicine"[Common Fields: Intelligent] OR "methotrexate"[Common Fields: Intelligent]) AND ("coronary heart disease"[Common Fields: Intelligent] OR "myocardial infarction"[Common Fields: Intelligent] OR "coronary artery disease"[Common Fields: Intelligent] OR "acute coronary syndrome"[Common Fields: Intelligent]) | 2557 |
| #2 | "anti-inflammatory agents"[Common Fields: Intelligent] OR "interleukin"[Common Fields: Intelligent] OR "colchicine"[Common Fields: Intelligent] OR "methotrexate"[Common Fields: Intelligent] | 108，990 |
| #1 | "coronary heart disease"[Common Fields: Intelligent] OR "myocardial infarction"[Common Fields: Intelligent] OR "coronary artery disease"[Common Fields: Intelligent] OR "acute coronary syndrome"[Common Fields: Intelligent] | 342，098 |

**Figure1 Risk of bias summary: review authors' judgements about each risk of bias item for each included study.**

**
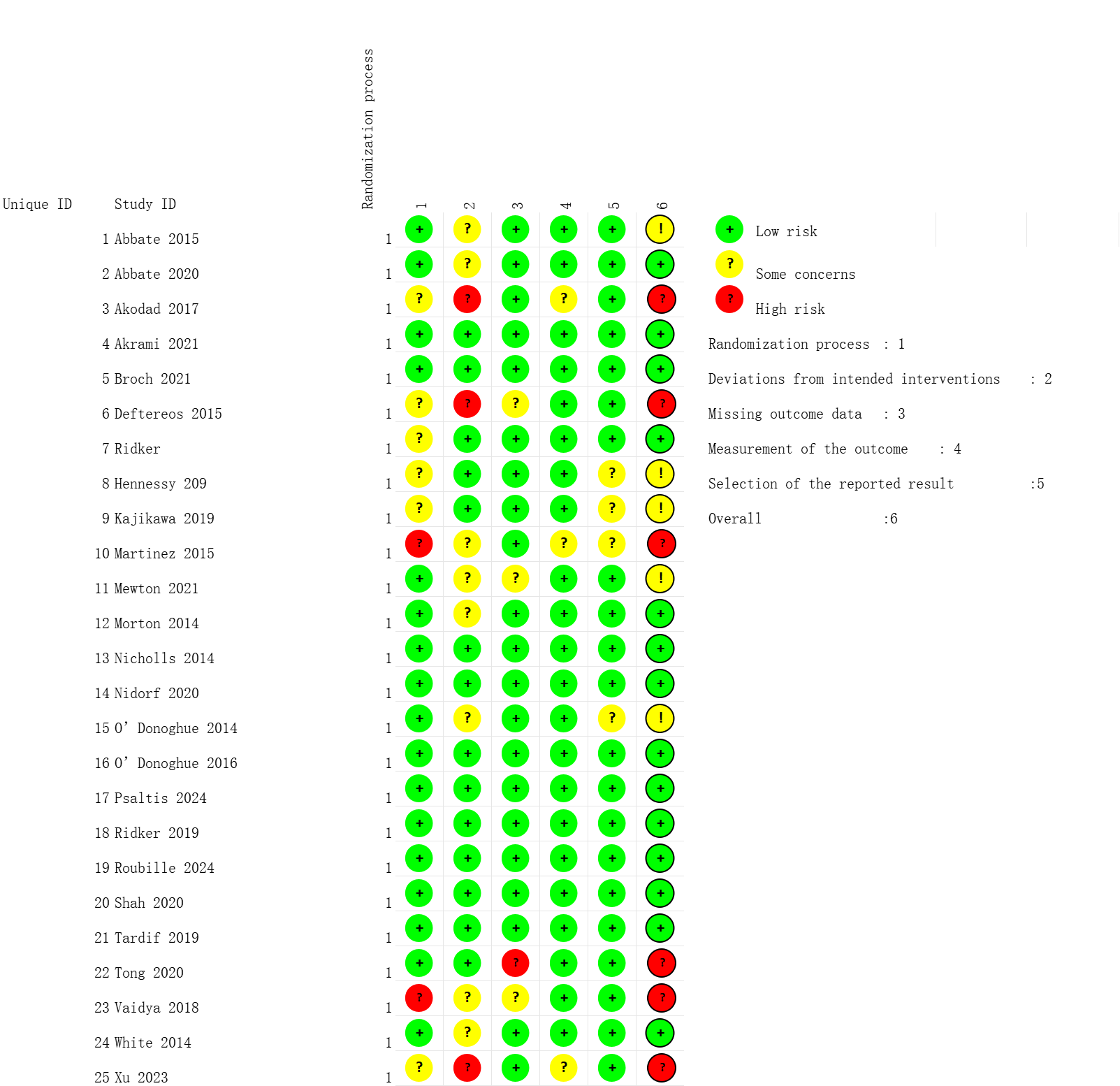
**

**Figure 2 1 Incidence of MACE, subgroup analysis of drug type**

**
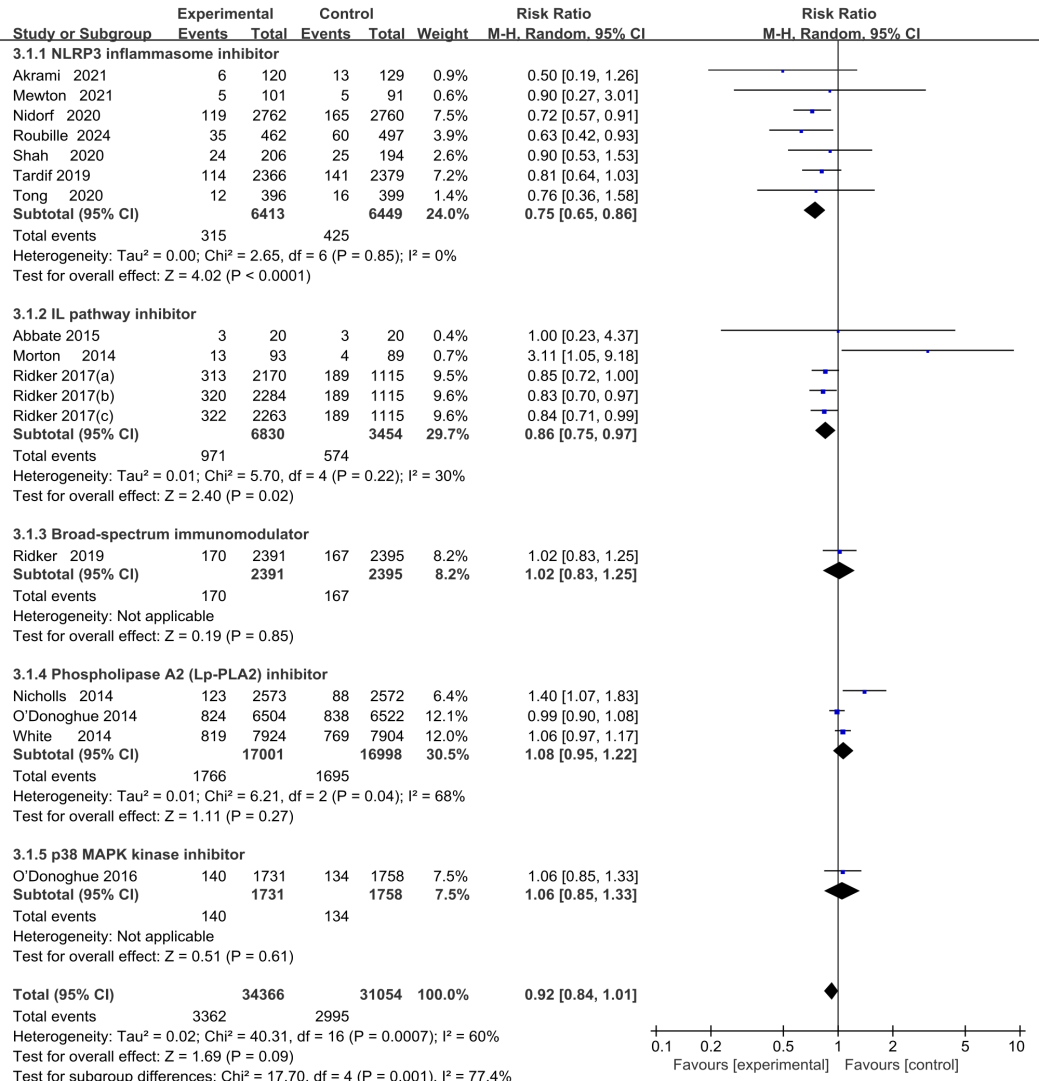
**

**MACE, major adverse cardiovascular events**

**Figure 2 2 Incidence of MACE, subgroup analysis of follow-up time**

**
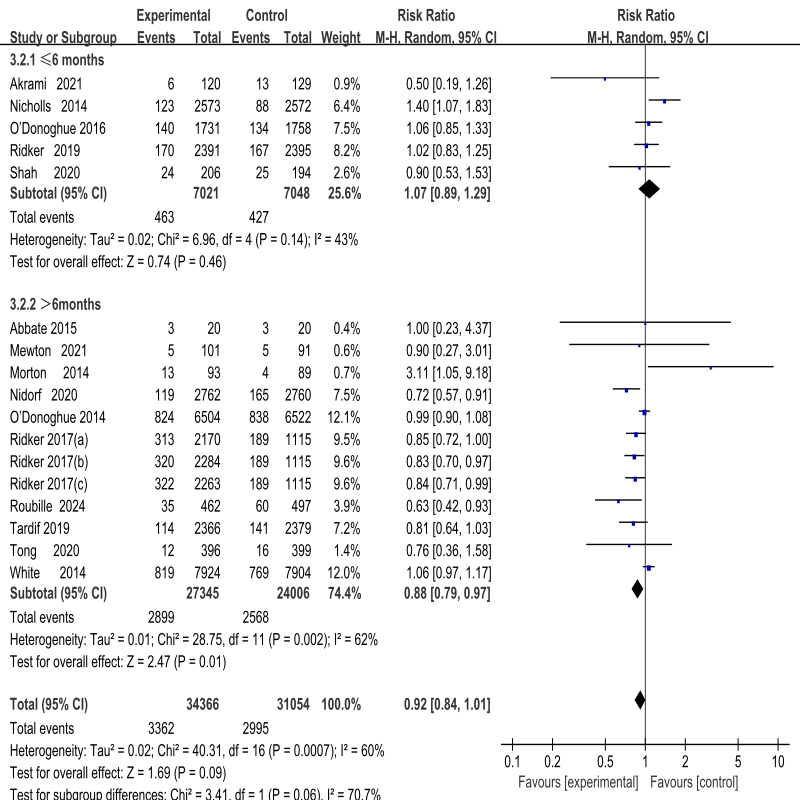
**

**Figure 2 3 Incidence of MACE, subgroup analysis of disease classification**

**
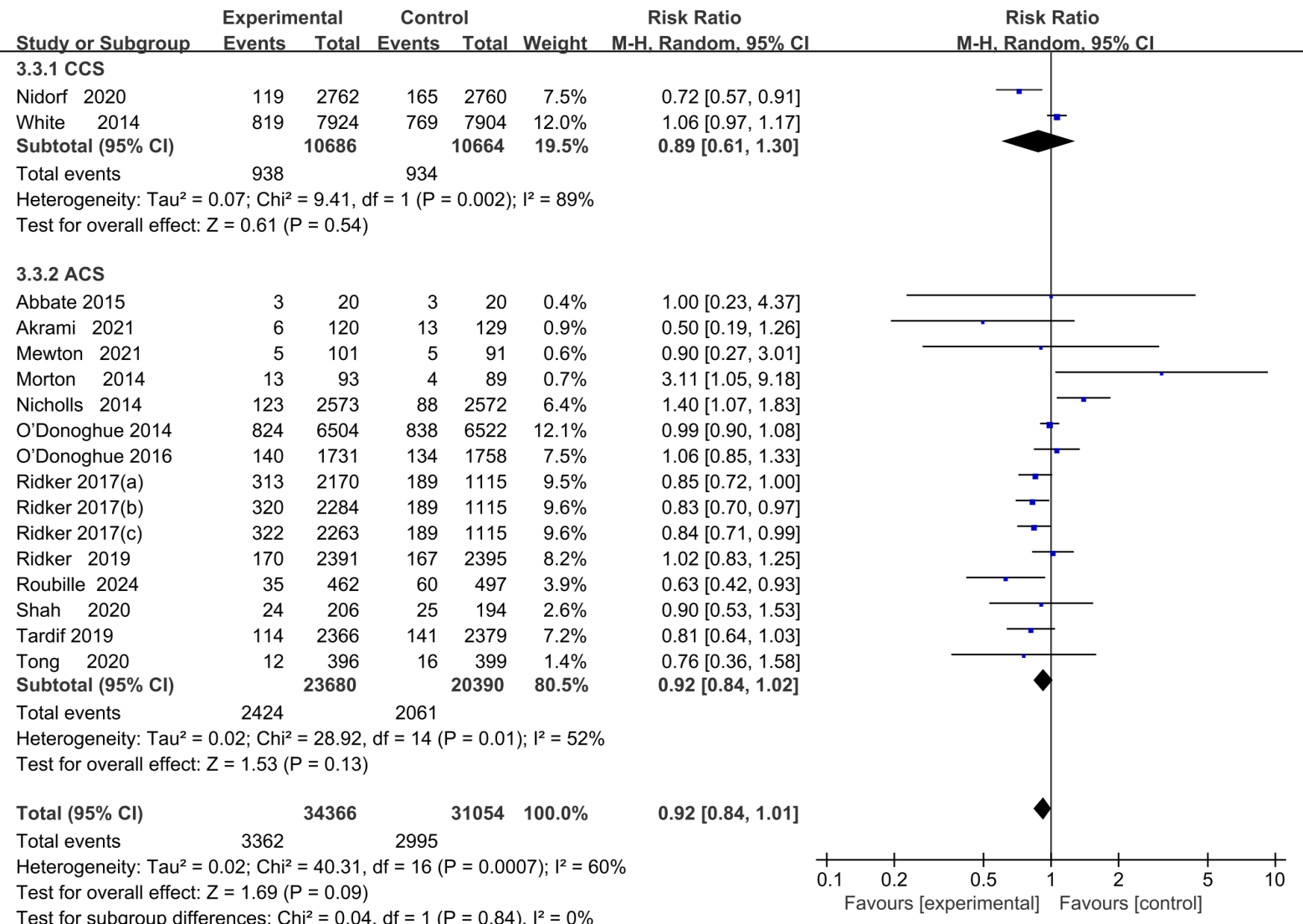
**

**Figure3 Incidence of CA**

**
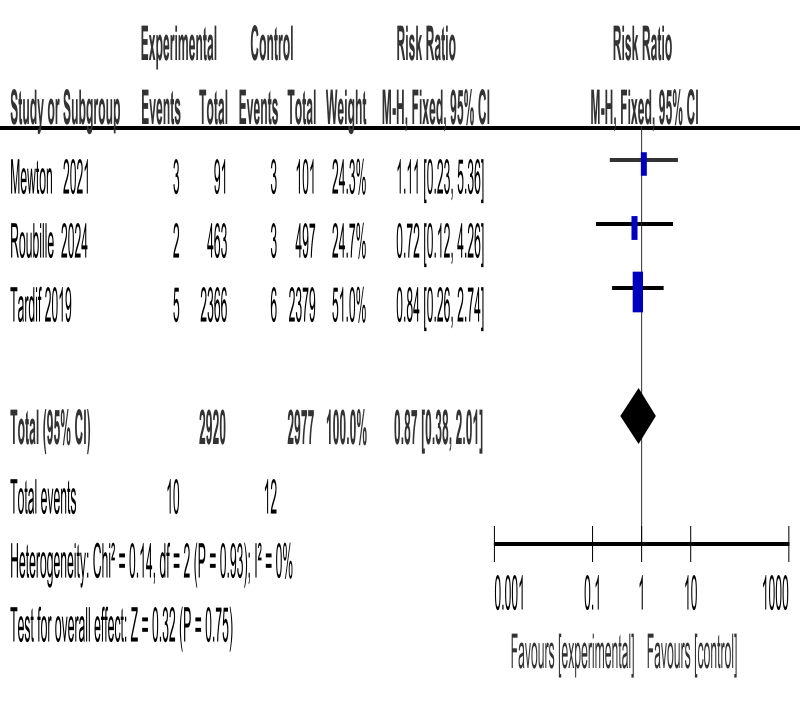
**

**CA: cardiac arrest.**

**Figure4** Incidence of angina

**
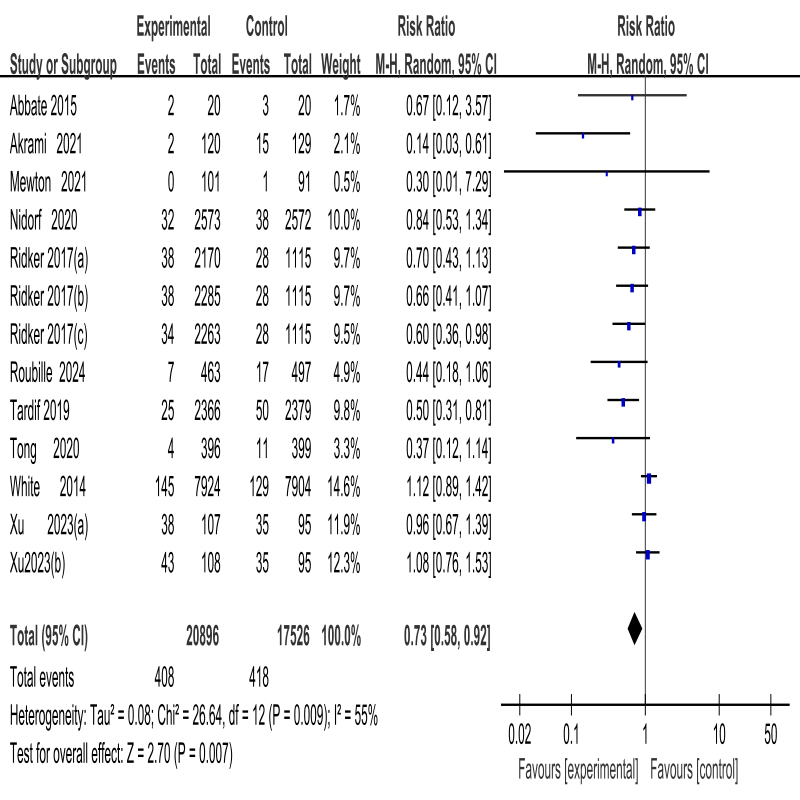
**

**Figure5 Incidence of all-cause mortality**

**
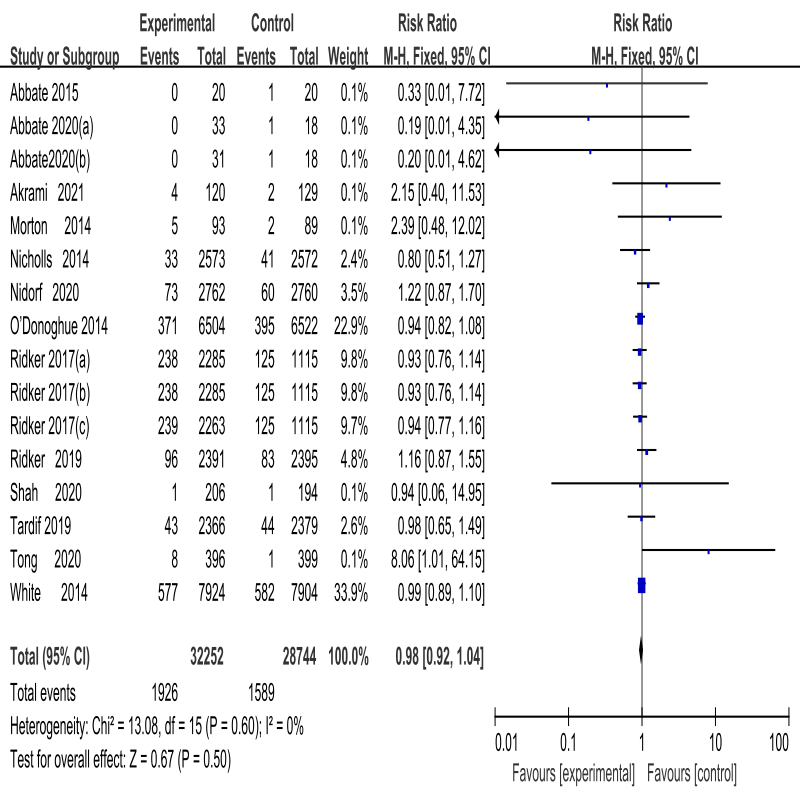
**

**Figure6 Incidence of revascularization**

**
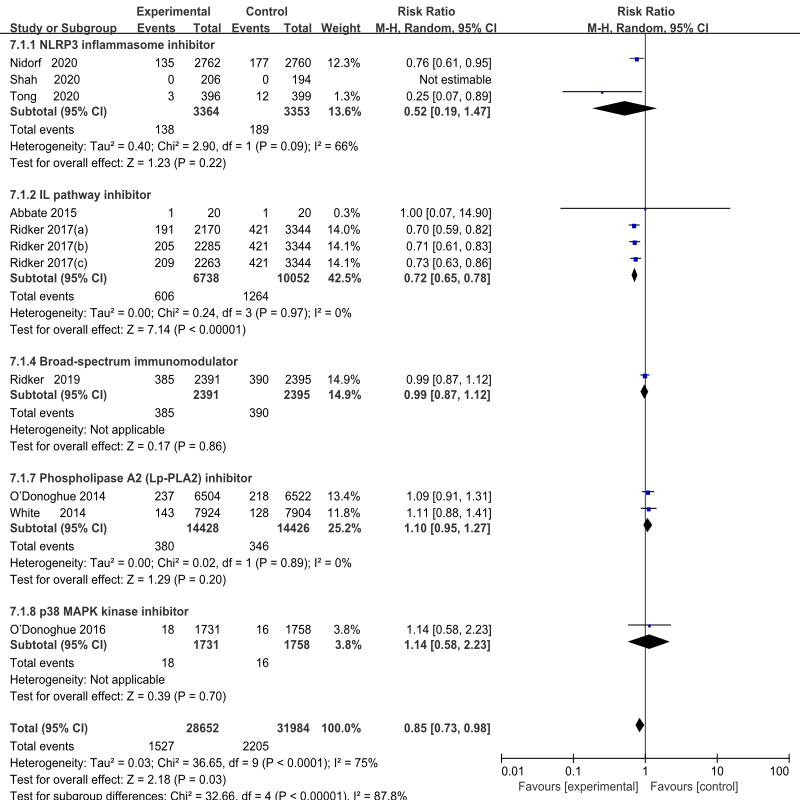
**

**Figure7 Incidence of gastrointestinal adverse effect**

**
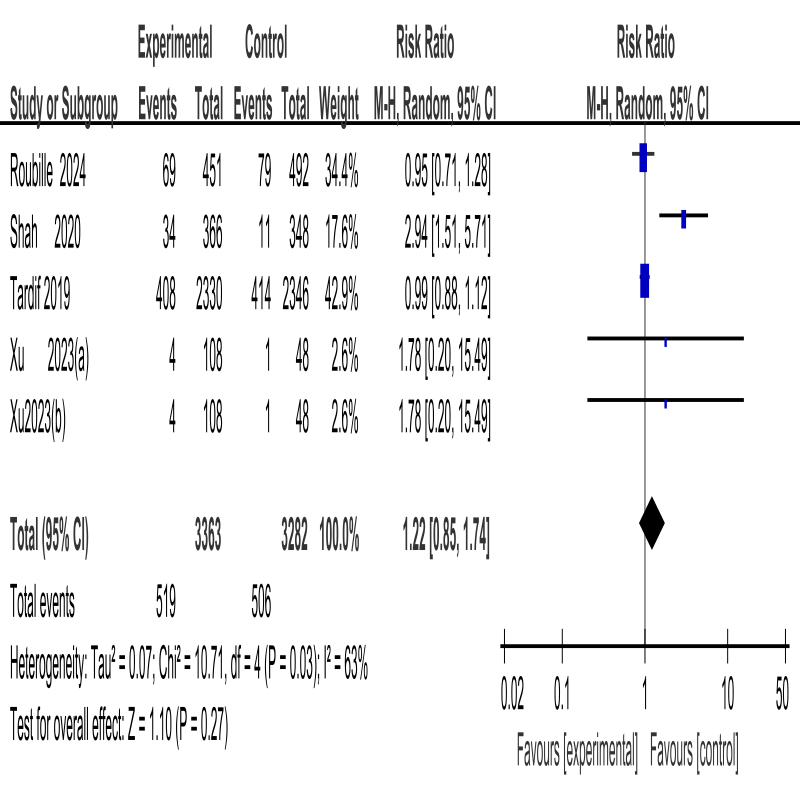
**

**Figure8 hs-CRP**

**
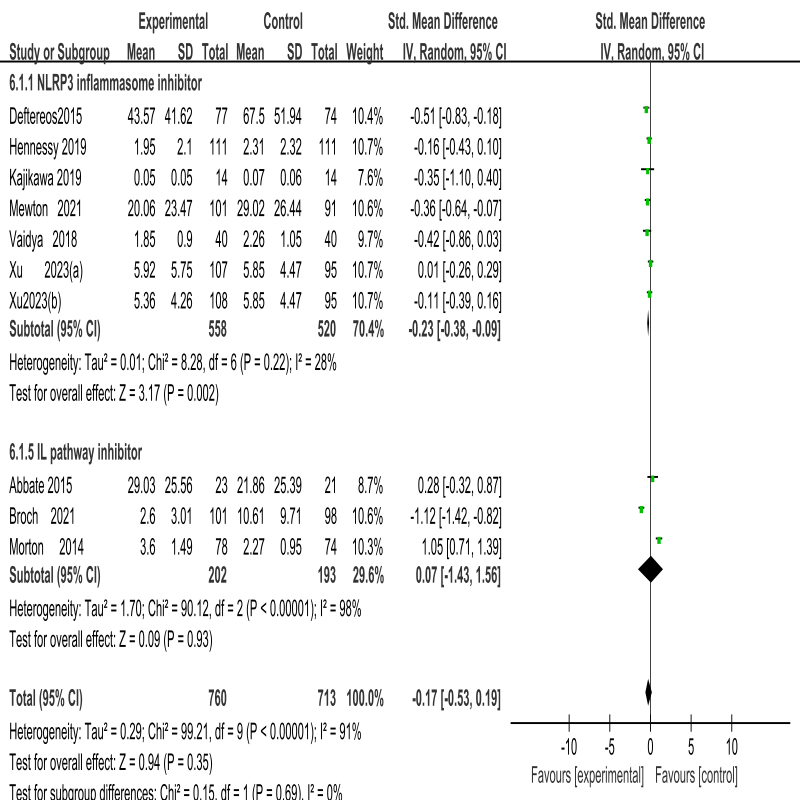
**

**hs-CRP, high-sensitivity C-reactive protein**

**Figure9 IL-6**

**
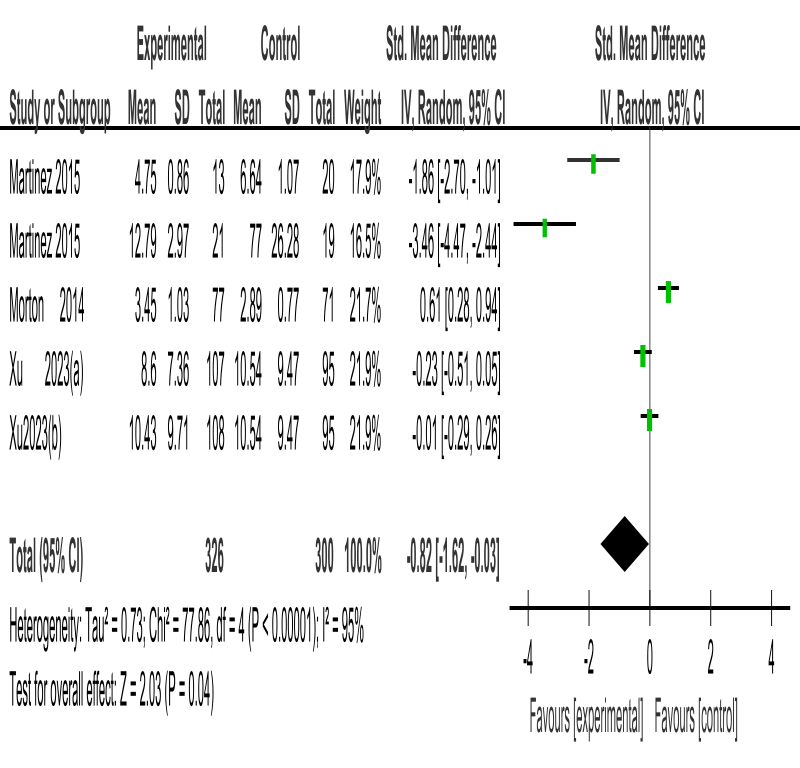
**

**IL, Interleukin**

**Figure10 neutrophil count.**

**
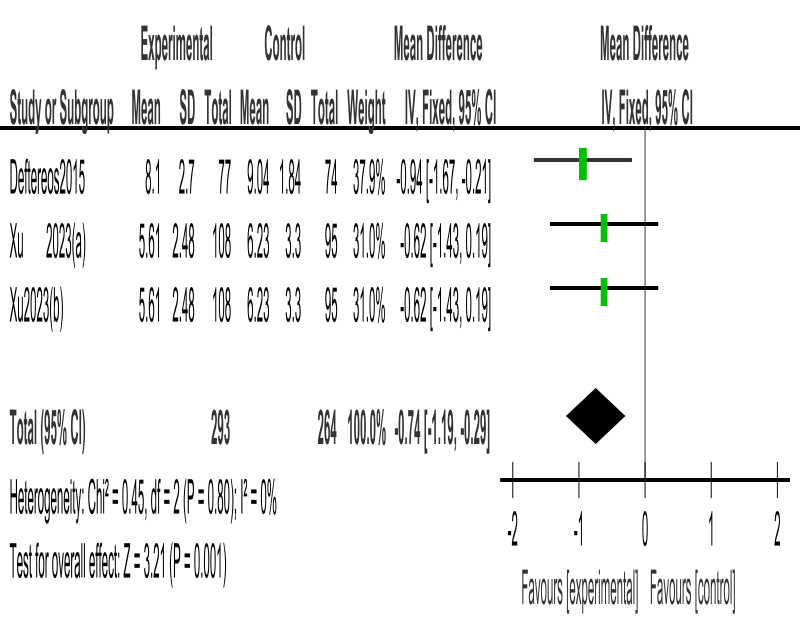
**

**Figure11 LVEF**

**
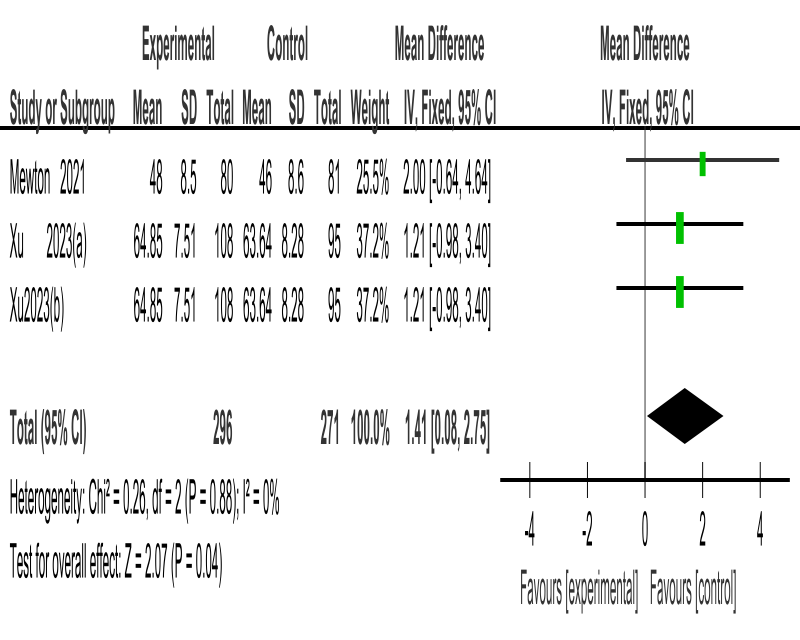
**

**LVEF, left ventricular ejection fraction**

**Figure 12 Incidence of infection**

**
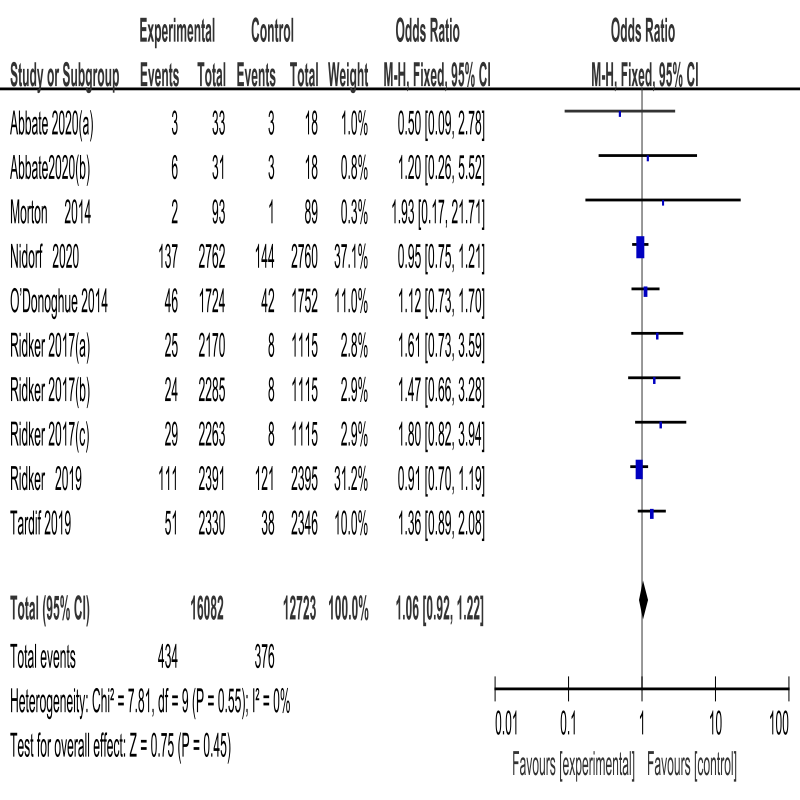
**

****Data of Dichotomous variable****

****1.****

| Incidence of MACE | Experimental | | Control | |
| --- | --- | --- | --- | --- |
| ID | Events | Total | Events | Total |
| Abbate 2015 | 3 | 20 | 3 | 20 |
| Akrami 2021 | 6 | 120 | 13 | 129 |
| Ridker 2017（1） | 321 | 2170 | 189 | 1115 |
| Ridker 2017（2） | 332 | 2285 | 189 | 1115 |
| Ridker 2017（3） | 340 | 2263 | 189 | 1115 |
| Mewton 2021 | 5 | 101 | 5 | 91 |
| Morton 2014 | 13 | 93 | 4 | 89 |
| Nicholls 2014 | 123 | 2573 | 88 | 2572 |
| Nidorf 2020 | 119 | 2762 | 165 | 2760 |
| O’Donoghue 2014 | 824 | 6504 | 838 | 6522 |
| O’Donoghue 2016 | 140 | 1731 | 134 | 1758 |
| Ridker 2019 | 170 | 2391 | 167 | 2395 |
| Roubille 2024 | 35 | 462 | 60 | 497 |
| Shah 2020 | 24 | 206 | 25 | 194 |
| Tardif 2019 | 114 | 2366 | 141 | 2379 |
| Tong 2020 | 12 | 396 | 16 | 399 |
| White 2014 | 819 | 7924 | 769 | 7904 |

****2.****

| Incidence of CA | Experimental | | Control | |
| --- | --- | --- | --- | --- |
| ID | Events | Total | Events | Total |
| Mewton 2021 | 3 | 91 | 3 | 101 |
| Roubille 2024 | 2 | 463 | 3 | 497 |
| Tardif 2019 | 5 | 2366 | 6 | 2379 |

**3.**

| Incidence of angina | Experimental | | Control | |
| --- | --- | --- | --- | --- |
| ID | Events | Total | Events | Total |
| Abbate 2015 | 2 | 20 | 3 | 20 |
| Akrami 2021 | 2 | 120 | 15 | 129 |
| Everett 2017（1） | 38 | 2170 | 28 | 1115 |
| Everett 2017（2） | 38 | 2285 | 28 | 1115 |
| Everett 2017（3） | 34 | 2263 | 28 | 1115 |
| Mewton 2021 | 0 | 101 | 1 | 91 |
| Nicholls 2014 | 32 | 2573 | 38 | 2572 |
| Roubille 2024 | 7 | 463 | 17 | 497 |
| Tardif 2019 | 25 | 2366 | 50 | 2379 |
| Tong 2020 | 4 | 396 | 11 | 399 |
| White 2014 | 145 | 7924 | 129 | 7904 |
| Xu 2023 (1) | 38 | 107 | 35 | 95 |
| Xu 2023 (2) | 43 | 108 | 35 | 95 |

**4.**

| Incidence of gastrointestinal | Experimental | | Control | |
| --- | --- | --- | --- | --- |
| ID | Events | Total | Events | Total |
| Roubille 2024 | 69 | 451 | 79 | 492 |
| Shah 2020 | 34 | 366 | 11 | 348 |
| Tardif 2019 | 408 | 2330 | 414 | 2346 |
| Xu 2023 （1） | 5 | 107 | 1 | 48 |
| Xu 2023 （2） | 4 | 108 | 1 | 48 |

**5.**

| Incidence of revascularization | Experimental | | Control | |
| --- | --- | --- | --- | --- |
| ID | Events | Total | Events | Total |
| Abbate 2015 | 1 | 20 | 1 | 20 |
| Ridker 2017（1） | 191 | 2170 | 140 | 1115 |
| Ridker 2017（2） | 205 | 2285 | 140 | 1115 |
| Ridker 2017（3） | 209 | 2263 | 140 | 1115 |
| Nidorf 2020 | 135 | 2762 | 177 | 2760 |
| O’Donoghue 2014 | 237 | 6504 | 218 | 6522 |
| O’Donoghue 2016 | 18 | 1731 | 16 | 1758 |
| Ridker 2019 | 385 | 2391 | 390 | 2395 |
| Shah 2020 | 0 | 206 | 0 | 194 |
| Tong 2020 | 3 | 396 | 12 | 399 |
| White 2014 | 143 | 7924 | 128 | 7904 |

**6.**

| Incidence of all-cause mortality | Experimental | | Control | |
| --- | --- | --- | --- | --- |
| ID | Events | Total | Events | Total |
| Abbate 2015 | 0 | 20 | 1 | 20 |
| Abbate 2019（1） | 0 | 33 | 1 | 35 |
| Abbate 2019（2） | 0 | 31 | 1 | 35 |
| Akrami 2021 | 4 | 120 | 2 | 129 |
| Ridker 2017(1) | 238 | 2285 | 125 | 1115 |
| Ridker 2017(2) | 238 | 2285 | 125 | 1115 |
| Ridker 2017(3) | 239 | 2263 | 125 | 1115 |
| Morton 2014 | 5 | 93 | 2 | 89 |
| Nicholls 2014 | 33 | 2573 | 41 | 2572 |
| Nidorf 2020 | 73 | 2762 | 60 | 2760 |
| O’Donoghue 2014 | 371 | 6504 | 395 | 6522 |
| Ridker 2019 | 96 | 2391 | 83 | 2395 |
| Shah 2020 | 1 | 206 | 1 | 194 |
| Tardif 2019 | 43 | 2366 | 44 | 2379 |
| Tong 2020 | 8 | 396 | 1 | 399 |
| White 2014 | 577 | 7924 | 582 | 7904 |

**7.**

| Incidence of infection | Experimental | | Control | |
| --- | --- | --- | --- | --- |
| ID | Events | Total | Events | Total |
| Ridker 2017（1） | 25 | 2170 | 8 | 1115 |
| Ridker 2017（2） | 24 | 2285 | 8 | 1115 |
| Ridker 2017（3） | 29 | 2263 | 8 | 1115 |
| Ridker 2019 | 111 | 2391 | 121 | 2395 |
| Abbate 2019（1） | 3 | 33 | 5 | 35 |
| Abbate 2019（2） | 6 | 31 | 5 | 35 |
| Morton 2014 | 2 | 93 | 1 | 89 |
| Nidorf 2020 | 137 | 2762 | 144 | 2760 |
| O’Donoghue 2014 | 46 | 1724 | 42 | 1752 |
| Tardif 2019 | 51 | 2330 | 38 | 2346 |

**Data of Continuous variable**

**1.**

| hs-CRP | Experimental | | | Control | | |
| --- | --- | --- | --- | --- | --- | --- |
| ID | Mean | SD | Total | Mean | SD | Total |
| Akodad 2017 | 29.03 | 25.56 | 23 | 21.86 | 25.39 | 21 |
| Broch 2021 | 2.60 | 3.01 | 101 | 10.61 | 9.71 | 98 |
| Deftereos2015 | 43.57 | 41.62 | 77 | 67.50 | 51.94 | 74 |
| Hennessy 2019 | 1.95 | 2.10 | 111 | 2.31 | 2.32 | 111 |
| Kajikawa 2019 | 0.05 | 0.05 | 14 | 0.07 | 0.06 | 14 |
| Mewton 2021 | 20.06 | 23.47 | 101 | 29.02 | 26.44 | 91 |
| Morton 2014 | 3.60 | 1.49 | 78 | 2.27 | 0.95 | 74 |
| Vaidya 2018 | 1.85 | 0.90 | 40 | 2.26 | 1.05 | 40 |
| Xu 2023（1） | 5.92 | 5.75 | 107 | 5.85 | 4.47 | 95 |
| Xu 2023（2） | 5.36 | 4.26 | 108 | 5.85 | 4.47 | 95 |

**2.**

| IL—6 | Experimental | | | Control | | |
| --- | --- | --- | --- | --- | --- | --- |
| 研究ID | Mean | SD | Total | Mean | SD | Total |
| Martine2015 ACS | 12.79 | 2.97 | 21 | 77 | 26.28 | 19 |
| Martine2015 CAD | 4.75 | 0.86 | 13 | 6.64 | 1.07 | 20 |
| Morton 2014 | 3.45 | 1.03 | 77 | 2.89 | 0.77 | 71 |
| Xu 2023（1） | 8.6 | 7.36 | 107 | 10.54 | 9.47 | 95 |
| Xu 2023（2） | 10.43 | 9.71 | 108 | 10.54 | 9.47 | 95 |

**3.**

| LVEF | Experimental | | | Control | | |
| --- | --- | --- | --- | --- | --- | --- |
| ID | Mean | SD | Total | Mean | SD | Total |
| Mewton 2021 | 48 | 8.5 | 80 | 46 | 8.6 | 81 |
| Xu 2023（1） | 63.35 | 8.26 | 107 | 63.64 | 8.28 | 95 |
| Xu 2023（2） | 64.85 | 7.51 | 108 | 63.64 | 8.28 | 95 |

**4.**

| neutrophil count | Experimental | | | Control | | |
| --- | --- | --- | --- | --- | --- | --- |
| ID | Mean | SD | Total | Mean | SD | Total |
| Deftereos2015 | 8.10 | 2.70 | 77 | 9.04 | 1.84 | 74 |
| Xu 2023（1） | 5.59 | 2.82 | 107 | 6.23 | 3.30 | 95 |
| Xu 2023（2） | 5.61 | 2.48 | 108 | 6.23 | 3.30 | 95 |

**PRISMA Checklist**

| **Section and Topic** | **Item #** | **Checklist item** | **Location where item is reported** |
| --- | --- | --- | --- |
| **TITLE** | | |  |
| Title | 1 | Identify the report as a systematic review. | Page 1, line 1-2. |
| **ABSTRACT** | | |  |
| Abstract | 2 | See the PRISMA 2020 for Abstracts checklist. | Page 3-4, line 45-79 |
| **INTRODUCTION** | | |  |
| Rationale | 3 | Describe the rationale for the review in the context of existing knowledge. | Page 4-6, line 81-112 |
| Objectives | 4 | Provide an explicit statement of the objective(s) or question(s) the review addresses. | Page 6, line 113-122 |
| METHODS | | |  |
| Eligibility criteria | 5 | Specify the inclusion and exclusion criteria for the review and how studies were grouped for the syntheses. | Page 7, line 137-147 |
| Information sources | 6 | Specify all databases, registers, websites, organisations, reference lists and other sources searched or consulted to identify studies. Specify the date when each source was last searched or consulted. | Page 6-7, line 123-127 |
| Search strategy | 7 | Present the full search strategies for all databases, registers and websites, including any filters and limits used. | Page 6-7, line 128-136,Supplementary table1 |
| Selection process | 8 | Specify the methods used to decide whether a study met the inclusion criteria of the review, including how many reviewers screened each record and each report retrieved, whether they worked independently, and if applicable, details of automation tools used in the process. | Page 7, line 148-154 |
| Data collection process | 9 | Specify the methods used to collect data from reports, including how many reviewers collected data from each report, whether they worked independently, any processes for obtaining or confirming data from study investigators, and if applicable, details of automation tools used in the process. | Page 8-9, line 162-179 |
| Data items | 10a | List and define all outcomes for which data were sought. Specify whether all results that were compatible with each outcome domain in each study were sought (e.g. for all measures, time points, analyses), and if not, the methods used to decide which results to collect. | Page 8, line 165-172 |
|  | 10b | List and define all other variables for which data were sought (e.g. participant and intervention characteristics, funding sources). Describe any assumptions made about any missing or unclear information. | Page 33-37, Table1. |
| Study risk of bias assessment | 11 | Specify the methods used to assess risk of bias in the included studies, including details of the tool(s) used, how many reviewers assessed each study and whether they worked independently, and if applicable, details of automation tools used in the process. | Page 7-8, line 154-160 |
| Effect measures | 12 | Specify for each outcome the effect measure(s) (e.g. risk ratio, mean difference) used in the synthesis or presentation of results. | Page 9, line 178-190 |
| Synthesis methods | 13a | Describe the processes used to decide which studies were eligible for each synthesis (e.g. tabulating the study intervention characteristics and comparing against the planned groups for each synthesis (item #5)). | Page 8, line 105-106 |
|  | 13b | Describe any methods required to prepare the data for presentation or synthesis, such as handling of missing summary statistics, or data conversions. | Page 10, line 121-122 |
|  | 13c | Describe any methods used to tabulate or visually display results of individual studies and syntheses. | Page 33-37, Table1. |
|  | 13d | Describe any methods used to synthesize results and provide a rationale for the choice(s). If meta-analysis was performed, describe the model(s), method(s) to identify the presence and extent of statistical heterogeneity, and software package(s) used. | Page 9, Line 189-190 |
|  | 13e | Describe any methods used to explore possible causes of heterogeneity among study results (e.g. subgroup analysis, meta-regression). | Page 9, Line 189-193, |
|  | 13f | Describe any sensitivity analyses conducted to assess robustness of the synthesized results. | Page 9, Line 193-195 |
| Reporting bias assessment | 14 | Describe any methods used to assess risk of bias due to missing results in a synthesis (arising from reporting biases). | Page 9, Line 195-196 |
| Certainty assessment | 15 | Describe any methods used to assess certainty (or confidence) in the body of evidence for an outcome. | Page 9-10, Line 197-202 |
| RESULTS | | |  |
| Study selection | 16a | Describe the results of the search and selection process, from the number of records identified in the search to the number of studies included in the review, ideally using a flow diagram. | Page 10, Line 204-209, Fig1 |
|  | 16b | Cite studies that might appear to meet the inclusion criteria, but which were excluded, and explain why they were excluded. | No study as this. |
| Study characteristics | 17 | Cite each included study and present its characteristics. | Page 10, Line 210-214, Table 1 |
| Risk of bias in studies | 18 | Present assessments of risk of bias for each included study. | Page 10-11, Line 215-229,figure2 |
| Results of individual studies | 19 | For all outcomes, present, for each study: (a) summary statistics for each group (where appropriate) and (b) an effect estimate and its precision (e.g. confidence/credible interval), ideally using structured tables or plots. | Page 11, Line 237-238; Page 12, Line 244-245  Page 12, Line 253-254  Page 12, Line 258-260  Table2 |
| Results of syntheses | 20a | For each synthesis, briefly summarise the characteristics and risk of bias among contributing studies. | Page 13-15, Line 253-316, Supplementary figure 3-11 |
|  | 20b | Present results of all statistical syntheses conducted. If meta-analysis was done, present for each the summary estimate and its precision (e.g. confidence/credible interval) and measures of statistical heterogeneity. If comparing groups, describe the direction of the effect. | Page 13-15, Line 253-316, Supplementary figure 3-11 |
|  | 20c | Present results of all investigations of possible causes of heterogeneity among study results. | Page 15-16, Line 324-325 |
|  | 20d | Present results of all sensitivity analyses conducted to assess the robustness of the synthesized results. | Page15-16, Line 320-324;Table3 |
| Reporting biases | 21 | Present assessments of risk of bias due to missing results (arising from reporting biases) for each synthesis assessed. | Page 15,Line 318-319,figure4 |
| Certainty of evidence | 22 | Present assessments of certainty (or confidence) in the body of evidence for each outcome assessed. | Page 16, Line 329-332; |
| DISCUSSION | | |  |
| Discussion | 23a | Provide a general interpretation of the results in the context of other evidence. | Page 17-18, Line 336-342 |
|  | 23b | Discuss any limitations of the evidence included in the review. | Page 19-20, Line 383-393 |
|  | 23c | Discuss any limitations of the review processes used. | Page 26, line 274-279 |
|  | 23d | Discuss implications of the results for practice, policy, and future research. | Page 20， Line 394-401 |
| OTHER INFORMATION | | |  |
| Registration and protocol | 24a | Provide registration information for the review, including register name and registration number, or state that the review was not registered. | Page 4, Line 76 |
|  | 24b | Indicate where the review protocol can be accessed, or state that a protocol was not prepared. | Page 4, Line 76 |
|  | 24c | Describe and explain any amendments to information provided at registration or in the protocol. | Page 7, line 87-88 |
| Support | 25 | Describe sources of financial or non-financial support for the review, and the role of the funders or sponsors in the review. | Page 2, Line 24-27 |
| Competing interests | 26 | Declare any competing interests of review authors. | Page 2 Line 29-30 |
| Availability of data, code and other materials | 27 | Report which of the following are publicly available and where they can be found: template data collection forms; data extracted from included studies; data used for all analyses; analytic code; any other materials used in the review. | Page 2,Line 40-41 |

*From:* Page MJ, McKenzie JE, Bossuyt PM, Boutron I, Hoffmann TC, Mulrow CD, et al. The PRISMA 2020 statement: an updated guideline for reporting systematic reviews. BMJ 2021;372:n71. doi: 10.1136/bmj.n71

For more information, visit: <http://www.prisma-statement.org/>
